# Maternal asthma and newborn DNA methylation

**DOI:** 10.1186/s13148-025-01858-4

**Published:** 2025-05-10

**Authors:** Casper-Emil Tingskov Pedersen, Thanh T. Hoang, Jianping Jin, Anna Starnawska, Raquel Granell, Hannah R. Elliott, Anke Huels, Heather J. Zar, Dan J. Stein, Yining Zhang, Herman T. den Dekker, Liesbeth Duijts, Janine F. Felix, Júlia Sangüesa, Mariona Bustamante, Maribel Casas, Martine Vrijheid, Latha Kadalayil, Faisal I. Rezwan, Hasan Arshad, John W. Holloway, Stefan Röder, Ana C. Zenclussen, Gunda Herberth, Nicklas Heine Staunstrup, Henriette Thisted Horsdal, Jonathan Mill, Eilis Hannon, iPSYCH-MINERvA Group, Isabella Annesi-Maesano, Giancarlo Pesce, Nour Baïz, Barbara Heude, Sahra Hosseinian-Mohazzab, Carrie V. Breton, Sophia Harlid, Justin Harbs, Magnus Domellof, Christina West, Edwina Yeung, Xuehuo Zeng, Wenche Nystad, Siri E. Håberg, Maria C. Magnus, Diana Schendel, Stephanie J. London, Klaus Bønnelykke

**Affiliations:** 1 Copenhagen Prospective Studies On Asthma in Childhood, Herlev and Gentofte Hospital, COPSAC, University of Copenhagen, Ledreborg Alle 34 Gentofte, 2820 Copenhagen, Denmark; 2 Division of Intramural Research, National Institute of Environmental Health Sciences (NIEHS), National Institutes of Health (NIH), MD A3-05, PO 12233, Research Triangle Park, NC 27709 USA; 3 Department of Pediatrics, Division of Hematology-Oncology, Department of Pediatrics, Baylor College of Medicine, Houston, TX USA; 4 Dan L. Duncan Comprehensive Cancer Center, Baylor College of Medicine, Houston, TX USA; 5 Cancer and Hematology Center, Texas Children’s Hospital, Houston, TX USA; 6 Department of Biomedicine, Aarhus University, 8000 Aarhus, Denmark; 7 The Lundbeck Foundation Initiative for Integrative Psychiatric Research, iPSYCH, Aarhus, Denmark; 8 Center for Genomics and Personalized Medicine, CGPM, and Center for Integrative Sequencing, iSEQ, Aarhus University, Aarhus, Denmark; 9 Department of Population Health Sciences, Bristol Medical School, University of Bristol, Oakfield House, Oakfield Grove, Bristol, BS8 2BN UK; 10 Medical Research Council Integrative Epidemiology Unit at the University of Bristol, Bristol, BS8 2BN UK; 11 Department of Epidemiology, Rollins School of Public Health, Emory University, Atlanta, GA USA; 12 Ganagarosa Department of Environmental Health, Rollins School of Public Health, Emory University, Atlanta, GA USA; 13 Department of Biostatistics and Bioinformatics, Rollins School of Public Health, Emory University, Atlanta, GA USA; 14 SAMRC Unit On Child & Adolescent Health, Dept of Paediatrics, University of Cape Town, Cape Town, South Africa; 15 The Generation R Study Group and Department of Pediatrics, Erasmus MC, University Medical Center Rotterdam, Rotterdam, The Netherlands; 16 Department of Neonatal and Pedicatric Intensive Care, Division of Neonatology, Erasmus MC, University Medical Center Rotterdam, Rotterdam, The Netherlands; 17 ISGlobal, Barcelona, Spain; 18 Universitat Pompeu Fabra (UPF), Barcelona, Spain; 19 CIBER Epidemiología y Salud Pública, Madrid, Spain; 20 School of Chemistry, Faculty of Engineering and Physical Sciences, University of Southampton, Southampton, UK; 21 Department of Computer Science, Aberystwyth University, University, Aberystwyth, SY23 3DB UK; 22 Faculty of Medicine, Human Development and Health, University of Southampton, Southampton, UK; 23 David Hide Asthma and Allergy Research Centre, Isle of Wight, UK; 24 Clinical and Experimental Sciences, Faculty of Medicine, University of Southampton, Southampton, UK; 25 NIHR Southampton Biomedical Research Centre, Southampton General Hospital, Southampton, UK; 26 Department of Environmental Immunology, Helmholtz Centre for Environmental Research–UFZ, Leipzig, Germany; 27 National Centre for Register-Based Research, Aarhus University, Aarhus, Denmark; 28 Department of Clinical & Biomedical Sciences, University of Exeter Medical School, University of Exeter, Exeter, UK; 29 Institute Desbrest of Epidemiology and Public Health, University of Montpellier and INSERM, Montpellier, France; 30 Inserm, INRAE, Center for Research in Epidemiology and StatisticS (CRESS), Université Paris Cité and Université Sorbonne Paris Nord, 75004 Paris, France; 31 Department of Population and Public Health Sciences, University of Southern California, Los Angeles, CA USA; 32 Department of Diagnostics and Intervention, Oncology, Umeå University, Umeå, Sweden; 33 Department of Clinical Sciences, Pediatrics, Umeå University, Umeå, Sweden; 34 Epidemiology Branch, Division of Population Health Research, Division of Intramural Research, Eunice Kennedy Shriver National Institute of Child Health and Human Development, Bethesda, MD 20817 USA; 35 Glotech Inc., 1801 Research Blvd #605, Rockville, MD 20850 USA; 36 Department of Chronic Diseases, Division of Mental and Physical Health, Norwegian Institute of Public Health, Oslo, Norway; 37 Centre for Fertility and Health, Norwegian Institute of Public Health, Skøyen, P.O. Box 222, 0213 Oslo, Norway; 38 MRC Integrative Epidemiology Unit at the University of Bristol, Oakfield House, Oakfield Grove, Bristol, BS8 2BN UK; 39 AJ Drexel Autism Institute, Drexel University, Philadelphia, USA

## Abstract

**Background:**

Prenatal exposure to maternal asthma may influence DNA methylation patterns in offspring, potentially affecting their susceptibility to later diseases including asthma.

**Objective:**

To investigate the relationship between parental asthma and newborn blood DNA methylation.

**Methods:**

Epigenome-wide association analyses were conducted in 13 cohorts on 7433 newborns with blood methylation data from the Illumina450K or EPIC array. We used fixed effects meta-analyses to identify differentially methylated CpGs (DMCs) and comb-p to identify differentially methylated regions (DMRs) associated with maternal asthma during pregnancy and maternal asthma ever. Paternal asthma was analyzed for comparison. Models were adjusted for covariates and cell-type composition. We examined whether implicated sites related to gene expression analyses in publicly available data for childhood blood and adult lung.

**Results:**

We identified 27 CpGs associated with maternal asthma during pregnancy at False Discovery Rate < 0.05 but none for maternal asthma ever. Two distinct CpGs were associated with paternal asthma. We observed 5 DMRs associated with maternal asthma during pregnancy 3 associated with maternal asthma ever and 13 DMRs associated with paternal asthma. Gene expression analysis using data in blood from 832 children and lung from 424 adults showed associations between identified DMCs using maternal asthma and expression of several genes, including *HLA* genes and *HOXA5,* previously implicated in asthma or lung function.

**Conclusion:**

Parental asthma, especially maternal asthma during pregnancy, may be associated with alterations in newborn DNA methylation. These findings might shed light on underlying mechanisms for asthma susceptibility.

**Supplementary Information:**

The online version contains supplementary material available at 10.1186/s13148-025-01858-4.

## Introduction

Asthma is the most common chronic disease in childhood, leading to decreased quality of life for affected families and large costs to society due reduced productivity and missed school days [1]. Childhood asthma is a highly heritable disease with parental asthma being the strongest known risk factor for asthma in the offspring and heritability estimates between 35 and 95% [2]. Nevertheless, a significant portion of the asthma risk in offspring remains unexplained by genetic factors alone [3] and early life risk factors related to the perinatal environment [4] and comorbidity factors [5] have been reported. Information on epigenetic markers such as DNA methylation collected at birth, might provide insight into the mechanisms of prenatal programming of childhood asthma. Furthermore, clinical studies have suggested that there are sex differences in vulnerability to asthma. Such differences may be related to sex-specific DNA methylation levels [6, 7]. Therefore, we speculated that sex-specific effects in the association of parental asthma with offspring methylation might be observed.

Large-scale meta-analyses of DNA methylation have demonstrated that epigenetic alterations at birth and later in childhood are associated with childhood asthma, suggesting a potential role of epigenetic mechanisms in asthma development [8–11]. Some studies have found that maternal asthma is a stronger risk factor for childhood asthma than paternal asthma [12], implying a potential prenatal programming effect on the oocyte or fetus from a maternal asthma-associated milieu, possibly through epigenetic mechanisms. However, the potential role of parental asthma and the difference between maternal and paternal effects on offspring methylation has not been well explored.

Here, our main objective was to perform a large-scale meta-analysis of maternal asthma active during pregnancy, hypothesizing that epigenetic effects could be stronger from intrauterine “exposure” to active maternal asthma. We additionally examined associations between DNA methylation and maternal asthma history in relation to DNA methylation from various blood sources at birth in more than 7000 newborns from 13 cohorts. Given the interest in paternal exposures on offspring health, we similarly analyzed associations with paternal asthma, hypothesizing that a higher number of differentially methylated CpGs in the maternal compared to the paternal analysis would further support a direct exposure effect on offspring DNA methylation. Further, given the suggestions in the asthma literature about sex-specific effects of impacts of parental asthma in offspring, we investigated the role of offspring sex in each of these analyses by performing sex-stratified analyses and interaction testing. We conducted our analyses by testing associations of DNA methylation from various blood sources at both individual CpG sites and at differently methylated regions (DMR). We evaluated the potential functional impact of findings by integrating gene expression data from blood and lung tissue.

## Methods

### Study design

The study used DNA methylation array data from cohorts within the pregnancy and childhood epigenetics (PACE) Consortium. PACE is an international consortium of cohorts with DNA methylation data available at birth, in childhood and/or in adolescence using either the Illumina450K array or Illumina EPIC arrays [13, 14]. We evaluated a maternal diagnosis of asthma (ever and during pregnancy) in relation to DNA methylation data from various blood sources from newborns in a total of 13 cohorts (Avon Longitudinal Study of Parents and Children [ALSPAC], Children’s Health Study [CHS], Drakenstein Child Health Study [DCHS], Etude des Déterminants pré et post natals du développement et de la santé de l’Enfant [EDEN], Generation R [GENR], INfancia y Medio Ambiente [INMA], Isle of Wight 3rd Generation Birth Cohort [IOWF2], Lifestyle and environmental factors and their influence on the newborn allergy risk [LiNA], Father and Child Cohort Study the Multigenerational Familial and Environmental Risk for Autism [MINERvA] sample within The Integrative Psychiatric Research (iPSYCH) cohort, Norwegian Mother and Child cohorts [MoBa1] and [MoBa2], The NorthPop Birth Cohort Study [NorthPop] and the Upstate KIDS study [UpstateKIDS]). We also conducted analyses examining paternal asthma versus paternal asthma never in 11 cohorts with this information (ALSPAC, CHS, EDEN, GENR, INMA, IOW, LiNA, MoBa1, MoBa2, NorthPop and UpstateKIDS). A full list of all cohorts including case and control numbers across models is given in Table S1, and cohort-specific study descriptions and inclusion criteria are given in supplementary material.

#### Asthma definitions

Asthma during pregnancy was defined by maternally self-reported asthma and/or use of asthma medication and/or a doctor’s diagnosis during gestation. For all but one cohort, the asthma diagnosis was identified using questionnaires, whereas for the MINERvA cohort an asthma diagnosis was based on registries. Asthma ever was defined as self-reported asthma ever and/or use of asthma medication ever. By our definition, individuals classified as having asthma in pregnancy are also included in analyses of ever asthma. More detailed phenotype definitions for each cohort are given in Supplementary material. Asthma during pregnancy was defined in seven cohorts (EDEN, IOWF2, MINERvA, MoBA1, MoBA2, NorthPop and UpstateKIDS) with available data for 3899 individuals.

#### Methylation data measurement, quality control and annotation

Methylation was assessed using either the Illumina 450 K BeadChip platform or the Illumina EPIC 850 K chip. For all cohorts, the minimum recommended DNA amount of 500 ng was provided to the laboratories running the 450 K or EPIC arrays. Cohorts individually performed quality control, normalization and analyses of untransformed *β* values. Cross-reactive probes, probes located on X and Y chromosomes as well as probes that overlapped with known SNPs were excluded after meta-analysis [15]. Methylation beta values were trimmed using the 3*IQR trimming method as has been done previously [16], where beta values three times the interquartile range below the 25th percentile or above the 75th percentile for each CpG were removed [17].

#### Annotation of DNA methylation sites

We used the gene annotation provided in the Illumina annotation files for both DNA methylation chips. All annotations use the human GRCh37/hg19 assembly.

#### Cohort-specific statistical analyses

Each cohort ran the association between asthma types and DNA methylation using robust linear regression. Covariates included infant sex, gestational age as a continuous measure, mode of delivery with two categories: vaginal delivery and cesarian section delivery, maternal age as a continuous measure and socioeconomic status (cohort-specific definition, but in general maternal education and income). In this analysis, we adjusted for maternal smoking during pregnancy in three categories: none, quit early in pregnancy and those who smoke across pregnancy. Prior work in PACE cohorts has shown that the greatest impact of maternal smoking is seen for smoking that is sustained across the pregnancy not in the approximately half of smokers who quit early in pregnancy [18, 19]. The MoBa study found no associations for smoking by the mother that ended before pregnancy [18]. Cohorts were adjusted for batch effects by using ComBat [20] or by including a batch covariate in their models. The MINERvA cohort adjusted for DNA methylation smoking score at birth as a surrogate for maternal smoking [21]. If a selection factor was employed, cohorts additionally adjusted for this, for instance if a cohort contained cases and controls selected based on a condition or characteristic (see cohort-specific description given in Supplementary Material). Maternal BMI was not available for all included cohorts and was thus not included as a covariate; to accommodate this, we performed a lookup in a large PACE meta-analysis of maternal BMI and found no overlap with our findings [22].

Cell-type composition was adjusted by including all 7 estimated proportions of cells using the cord blood reference panel [23] calculated by the Houseman method [24] using the FlowSorted.Blood package available for *minfi* [25].

#### Meta-analyses

We meta-analyzed study-specific results with inverse variance weighting in METAL [26]. The meta-analysis was redone by an independent group using the same method and the results were compared to minimize the likelihood of human error. For the sex-stratified analyses, we restricted the meta-analyses to studies where there were at least 15 newborns of each sex exposed to the parental asthma condition under study (see Table S1) and used the resulting number of studies in the meta-analysis where sex and parental asthma were investigated using an interaction term. For maternal asthma ever, we used maternal asthma during pregnancy as a surrogate for the MINERvA and EDEN cohorts. We performed analyses restricted to either the 450 K chip including CpGs, after QC filtering, that overlapped between 450 K and the EPIC chip (424,403 CpGs) or were exclusive to the EPIC chip (321,034 CpGs). In total 10,922 CpGs were removed from the 450 K analysis because they were not captured on the EPIC chip. We restricted the analysis to CpGs with available data from at least three studies for probes on the 450 K chip and two studies for probes available on the EPIC chip data (as we only included two studies with such data) and accounted for multiple testing by controlling the false discovery rate (FDR) using a threshold of 5% for each chip-specific analysis along with a more strict FDR threshold of 0.025 as some would argue for using this threshold when separating CpGs into chip-specific analyses. We calculated if the observed effect sizes were homogeneous (I^2^ value) across cohorts using METAL [23]. We show forest plots for significantly differentially methylated CpGs (DMCs) including effect estimates and 95% confidence intervals for each cohort.

#### Analyses of differentially methylated regions

We identified differentially methylated regions (DMRs) using comb-p [27] as this method tends to be more conservative than DMRcate [28]. Comb-p corrects multiple comparisons through a one-step Šidák correction [27]. We identified significant DMRs using an adjusted FDR *p* value below 0.05, required at least three probes with a maximum distance of 500 bp. DMRs were annotated to the nearest gene, regulatory regions and proximity to CpG islands if present on the Illumina annotation file for the hg19 reference genome.

#### Identification of drug targets and mQTL associations using ChEMBL and GoDMC

We looked in the ChEMBL database (version 31, https://www.ebi.ac.uk/chembl/) to identify genes implicated in our analyses of DMCs or DMRs that previously have been targets of approved drugs or drugs in development. We did lookup of DMCs in the GoDMC database (http://mqtldb.godmc.org.uk/search) to look for potential methylation quantitative loci (mQTL) associated with asthma.

#### Correlation of DNA methylation and gene expression

We examined if DNA methylation at significant individual or DMR CpGs was related to gene expression using lookup in 39,749 significant expression quantitative trait methylation (eQTM) pairs (FDR < 0.05) of blood DNA methylation probes from the 450 K chip array and blood gene expression data from 832 children available in the HELIX consortium [29]. eQTMs were identified using linear regression of methylation levels in relation to expression at nearby genes (using a 1mb window centered on the TSS) [29]. In addition, we also included 8,646 significant eQTM pairs (and FDR < 0.05) of DNA methylation probes from the EPIC chip array and gene expression data from adult blood and lung tissue from 424 individuals in GTEx (https://www.gtexportal.org/home/). We did this by lookup in the summary statistic data from the cell-type-adjusted HELIX data and GTEx data separately and considered significance based on FDR *p* values below 0.05.

#### Data availability

Genome-wide meta-analysis results will be available in the following link upon publication: 10.5281/zenodo.13219057.

## Results

### Demographic description

There were 3899 individuals across 7 cohorts available for maternal asthma during pregnancy and 7433 individuals across 13 cohorts for maternal asthma ever. Supplementary material contains cohort-specific asthma definitions and distributions of parental asthma phenotypes. The prevalence of self-reported maternal asthma ranged from 2 to 35%, sustained smoking ranged between 0.4 and 30%. Most cohorts used the 450 K chip. Two cohorts (NorthPop and UpstateKIDS) had DNA methylation measured using the EPIC chip. Participants were primarily of European descent (Table 1). We did not see any overlap between our findings and a recently published EWAS on maternal BMI [22].

**Table 1:** Characteristics of participating study cohorts

Cohort	Tissue	Chip	Ethnicity	Male %	Sustained smoking during pregnancy, (%)	Maternal asthma during pregnancy, *N* (%)	Maternal asthma ever, *N* (%)	Paternal asthma ever, *N* (%)			
						Total *N*: 3899	Total *N*: 7433	Total *N*: 4919			
						Case *N* (%)	N	Case *N* (%)	*N*	Case *N* (%)	*N*
ALSPAC	Cord blood	450 K	EUR	48.5	9.4	NA	NA	84 (10)	841	58 (7)	815
CHS	Newborn blood spots	450 K	EUR	40.9	7.6	NA	NA	19 (9)	210	12 (6)	210
DCHS	Cord blood	450 K	African/Admixed	55.7	29.4	NA	NA	7 (3)	262	NA	NA
EDEN	Cord blood	450 K	EUR	60.2	24.2	17 (11)	161	17 (11)	161	12 (7)	161
GENR	Cord blood	450 K	EUR	48.6	10.8	NA	NA	57 (7)	868	53 (6)	868
INMA	Cord blood	450 K	EUR	51.6	14.4	NA	NA	31 (8)	376	28 (7)	376
IOW	Cord blood	450 K	EUR	51.1	24.5	19 (21)	90	35 (35)	99	23 (28)	82
LINA	Cord blood	450 K	EUR	52.7	14.8	NA	NA	50 (11)	456	38 (9)	439
MoBA1	Cord blood	450 K	EUR	53.3	15.2	85 (8)	1001	124 (12)	1040	64 (8)	776
MoBA2	Cord blood	450 K	EUR	56.5	12.2	51 (8)	637	74 (11)	660	50 (11)	452
MINERvA	Newborn peripheral blood	450 K	EUR	49.9	NA	30 (2)	1259	30 (2)	1259	NA	NA
NorthPop	Cord blood	EPIC	EUR	47.6	0.4	49 (22)	220	105 (16)	672	41 (19)	211
UpstateKIDS	Blood spots	EPIC	EUR. African	50.9	4.1	27 (5)	531	93 (18)	529	61 (12)	529

**Fig. 1:**
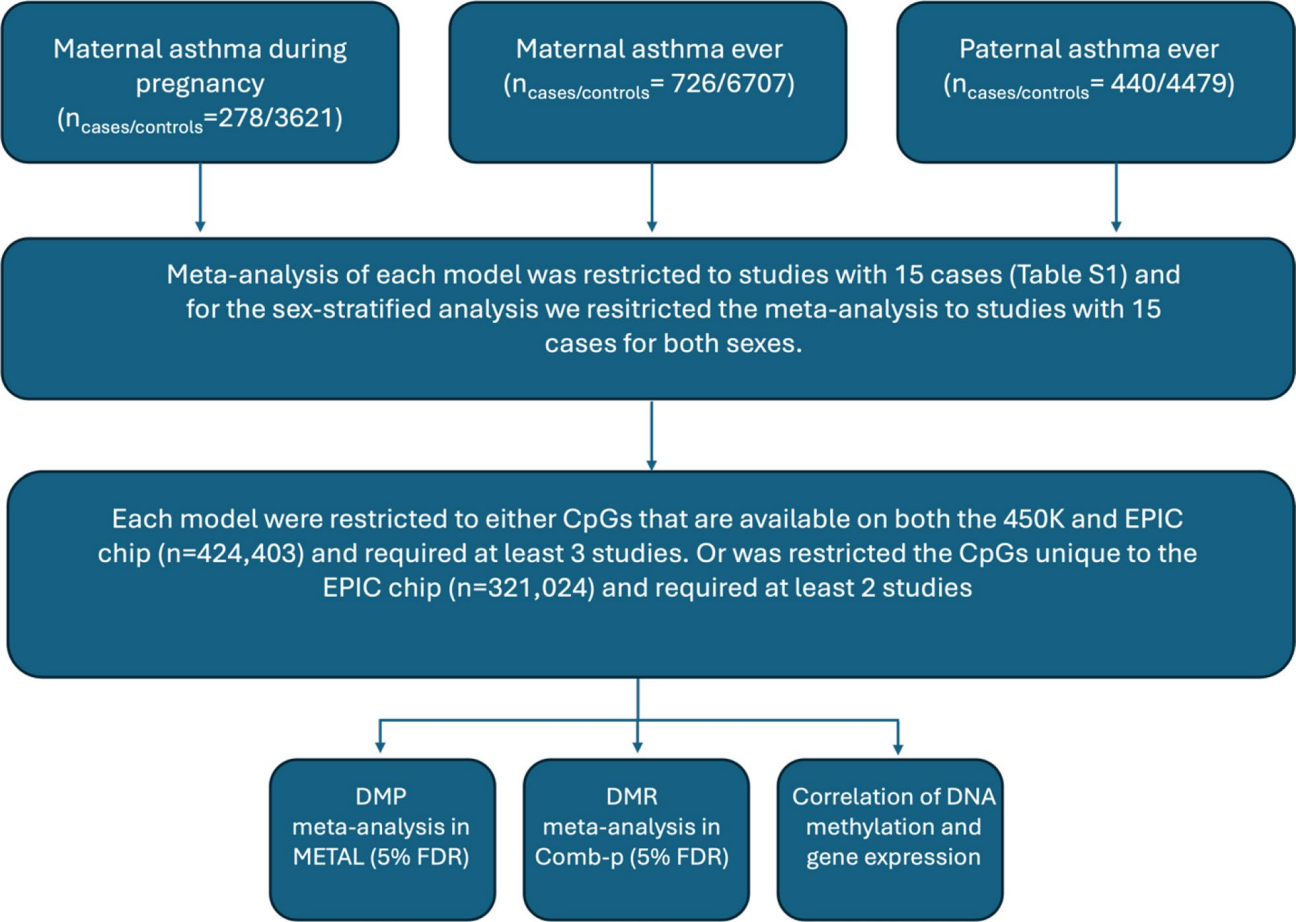
Flowchart describing the number of samples, the primary quality control filters as well as the analyses included

An overview of included analyses, primary quality control filters and analyses are shown in Fig. 1.

### Maternal asthma during pregnancy and newborn DNA methylation

**Fig. 2:**
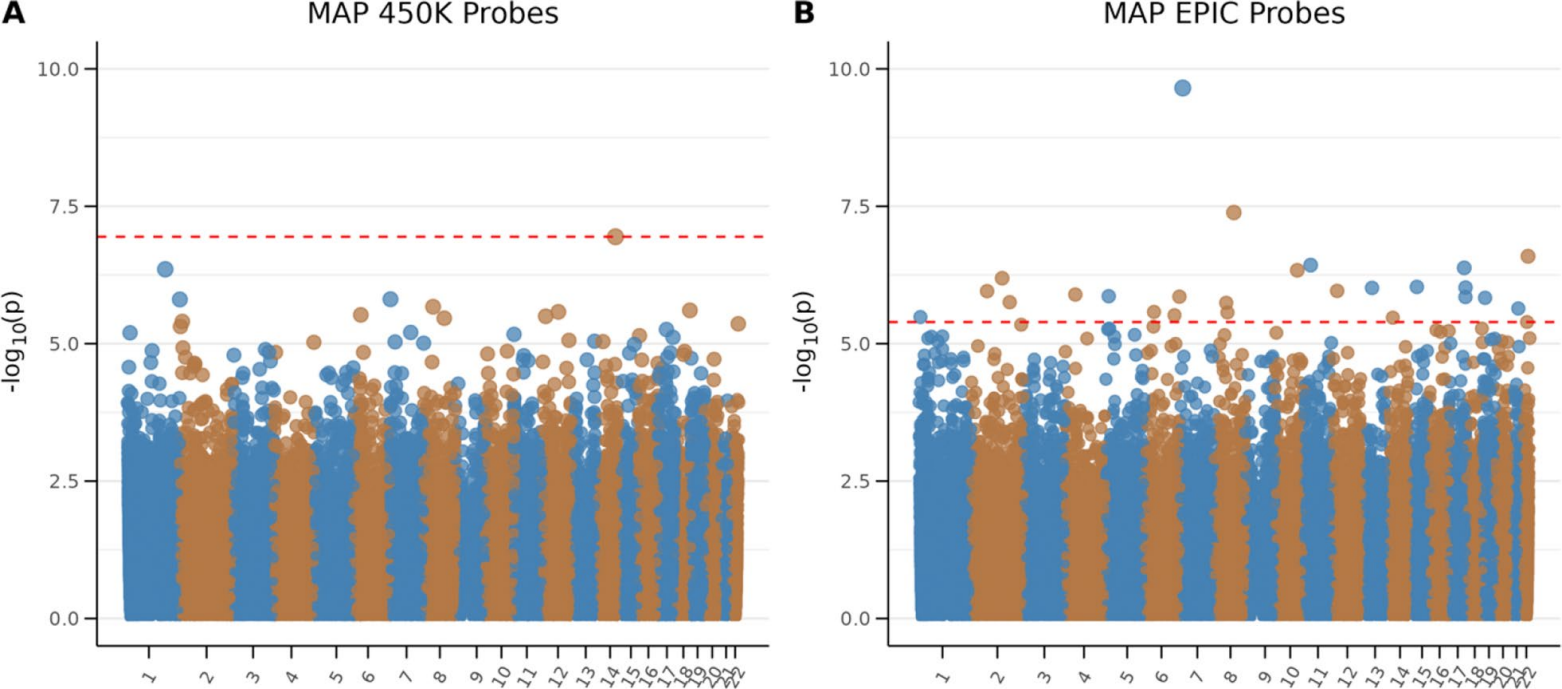
Manhattan plot of epigenome-wide association of maternal asthma during pregnancy and offspring methylation. Models include the following covariates: child sex, maternal smoking during pregnancy, gestational age, mode of delivery, maternal age at childbirth, maternal socioeconomic status, estimated cell type, batch covariates and ancestry. Panel A) shows analysis restricted to 435,329 probes on the 450 K chip for 7 cohorts using 202 exposed and 2946 non-exposed individuals (lambda = 1.09), and panel B) shows 321,034 probes exclusive to the EPIC chip for 2 cohorts using 76 exposed and 675 non-exposed individuals (lambda = 1.18). The red line indicates 5% FDR significance

The meta-analysis of newborn DNA methylation in relation to maternal asthma during pregnancy included 278 exposed and 3621 non-exposed participants from 7 cohorts: IOW, EDEN, MoBA1, MoBA2, NorthPop, UpstateKIDS and MINERvA. We identified 1 significant DMC for the 450 K chip (*λ* = 1.09), cg26963854 within the south shelf of a CpG island, which was not annotated to a specific gene (FDR 5%). We also identified 26 DMCs exclusive to the EPIC chip (FDR < 5%) using 76 exposed and 675 non-exposed participants from NorthPop and UpstateKIDS (*λ* = 1.18) (Fig. 2, Table 2). Using a stricter *P* value threshold of (FDR < 2.5%), we observe 6 DMCs pertaining only to the EPIC chip (Table S2). We identified 5 DMRs (encompassing 28 CpGs) in relation to maternal asthma during pregnancy for the 450 K chip and none for probes exclusive to the EPIC chip Table S3). Forest plots with cohort-specific beta values and 95% confidence intervals for the identified CpGs are shown in Fig. S1. For the CpG site (cg26963854) identified using the 450 K chip, the effect estimate was lower in the MINERvA cohort compared to that of the other included cohorts with the 450 K data, but without evidence of heterogeneity (P_heterogeneity_ = 0.14). For the EPIC chip, we saw no evidence of heterogeneity (Fig. S1).

**Table 2:** Twenty-seven differentially methylated CpGs (FDR < 0.05) from the meta-analysis of maternal asthma during pregnancy in relation to newborn methylation

CpG Name	Chr	Pos	UCSC Gene	UCSC group	Relation to CpG Island	Direction	Effect	SE	HetISq (%)	*P* value	FDR *P* value	Chip
cg26963854	14	95,902,398			S_Shelf	+ + − + + + +	0.0032042	0.0006041	37	1.13E-07	4.78E-02	450 K
cg08408040	7	11,381,133			OpenSea	− −﻿	− 0.0806398	0.0127099	75.4	2.23E-10	7.06E-05	EPIC
cg05760515	8	93,044,577	*RUNX1T1*	Body;5’UTR	OpenSea	+ +	0.0035094	0.0006397	0	4.11E-08	6.50E-03	EPIC
cg16664062	22	43,090,408	*A4GALT*	5’UTR	S_Shore	+ +	0.0104421	0.0020266	16.3	2.57E-07	2.44E-02	EPIC
cg01344764	11	31,979,230			OpenSea	− −﻿	− 0.0132207	0.0026012	0	3.73E-07	2.44E-02	EPIC
cg06909301	17	71,593,664	*SDK2*	Body	OpenSea	− +	− 0.003653	0.0007218	0	4.18E-07	2.44E-02	EPIC
cg01093036	10	104,489,529	*SFXN2*	Body	OpenSea	+ +	0.0087239	0.0017305	0	4.62E-07	2.44E-02	EPIC
cg00640622	2	144,020,044	*ARHGAP15*	Body	OpenSea	− +	− 0.0163449	0.0032839	79.2	6.45E-07	2.73E-02	EPIC
cg20264088	15	41,166,515	*RHOV*	TSS200	Island	+ +	0.0313048	0.0063805	40.4	9.28E-07	2.73E-02	EPIC
cg27290786	17	77,891,951	*LOC101928738; LOC101928766*	TSS1500;Body	OpenSea	+ +	0.0079921	0.0016307	0	9.53E-07	2.73E-02	EPIC
cg15066822	13	52,352,312	*DHRS12*	Body	OpenSea	+ +	0.0042652	0.0008708	0	9.68E-07	2.73E-02	EPIC
cg01573661	12	21,819,734			OpenSea	− −﻿	− 0.0137664	0.0028247	36.8	1.10E-06	2.73E-02	EPIC
cg09103231	2	73,182,547	*SFXN5*	Body	OpenSea	+ −	0.0064028	0.0013143	29	1.11E-06	2.73E-02	EPIC
cg15075638	4	47,814,806	*CORIN*	Body	OpenSea	− −﻿	− 0.0050016	0.0010327	0	1.28E-06	2.73E-02	EPIC
cg03443357	5	14,168,374	*TRIO*	Body	OpenSea	− −﻿	− 0.0108232	0.0022411	0	1.37E-06	2.73E-02	EPIC
cg02603747	6	166,460,087			OpenSea	− −﻿	− 0.0149353	0.0030951	0	1.40E-06	2.73E-02	EPIC
cg16446317	17	77,490,891	*RBFOX3*	5’UTR	OpenSea	+ +	0.0062025	0.0012861	0	1.42E-06	2.73E-02	EPIC
cg05590737	19	10,829,803	*DNM2*	Body	S_Shore	− −﻿	− 0.0187622	0.0038958	0	1.47E-06	2.73E-02	EPIC
cg13974400	2	179,637,873	*TTN*	Body	OpenSea	+ +	0.0023148	0.0004844	0	1.76E-06	3.01E-02	EPIC
cg10678257	8	56,987,912	*SNORD54;RPS20*	TSS1500	S_Shore	− −﻿	− 0.0061053	0.0012789	0	1.81E-06	3.01E-02	EPIC
cg16835843	21	45,363,997	*AGPAT3*	5’UTR	S_Shore	+ +	0.0085476	0.0018086	0	2.29E-06	3.62E-02	EPIC
cg20700171	6	46,729,036			OpenSea	− −﻿	− 0.0044141	0.0009398	0	2.65E-06	3.89E-02	EPIC
cg18940887	8	62,800,933			OpenSea	− −﻿	− 0.0076541	0.0016314	20.7	2.71E-06	3.89E-02	EPIC
cg04356230	6	143,251,273	*HIVEP2*	5’UTR	S_Shelf	− −﻿	− 0.0188473	0.0040371	7.4	3.03E-06	4.17E-02	EPIC
cg05492840	1	8,254,431			OpenSea	− −﻿	− 0.0170443	0.0036632	0	3.28E-06	4.26E-02	EPIC
cg09724019	14	35,192,414			OpenSea	− −﻿	− 0.0172851	0.0037194	36.2	3.36E-06	4.26E-02	EPIC
cg00128361	22	38,245,362	*EIF3L*	TSS200	Island	− −﻿	− 0.0024848	0.000539	0	4.04E-06	4,91E-02	EPIC

Results are separated by chip and an 5% FDR threshold was done for sites unique to either chip, respectively. None of the CpGs was located in an enhancer

For each cohort included in analysis, a + indicates a positive direction of effect while a − indicates a negative direction of effect. The order of cohorts is as follows: EDEN, IOW, MINERvA, MoBa1, MoBa2, NorthPop and UpstateKIDS

### Maternal asthma ever and newborn DNA methylation

The meta-analysis of newborn methylation and maternal asthma ever included 726 exposed and 6707 non-exposed participants from 13 cohorts: ALSPAC, CHS, DCHS, EDEN, GENR, INMA, IOW, LiNA, MINERvA, MoBA1, MoBA2, NorthPop and UpstateKIDS, and identified no associated probes (FDR < 0.05) for either the 450 K or EPIC chip (Fig. S2. We identified 3 DMRs (comprising 28 CpGs) for the 450 K chip but none for the EPIC chip (Table S4). We did not observe any significant probes (FDR < 0.05) if we restrict the maternal asthma ever analysis to the 7 cohorts in the maternal asthma during pregnancy analysis.

### Paternal asthma and newborn DNA methylation

The meta-analysis of newborn methylation and paternal asthma ever included 440 exposed and 4479 non-exposed participants from 9 cohorts: ALSPAC, GENR, INMA, IOW, LiNA, MoBA1, MoBA2, NorthPop and UpstateKids, and identified no probes associated with paternal asthma (FDR < 0.05) for the 450 K chip and 2 associated probes for the EPIC chip, namely cg08311378 in the gene body of *RPS6KA2* and cg07462855 in the gene body of *FAM160B1* (Fig. S3, Table S5)*.*These DMCs were not among the 26 DMCs identified using the EPIC chip for the analysis of maternal asthma during pregnancy nor were they at least nominally significant in the maternal asthma (active or ever) analysis. Forest plots for the identified DMCs are in Fig. S4.

We identified 11 DMRs encompassing 99 CpGs in relation to paternal asthma ever diagnosis for the 450 K and 2 for the EPIC chip, encompassing 9 CpGs (Tables S6). Some overlap was detected with DMRs associated with maternal pregnancy asthma status (in gene *PPT2; PRRT1*) and maternal ever asthma (*HOXA* genes).

### Correlation analysis of newborn methylation across maternal and paternal asthma

**Fig. 3:**
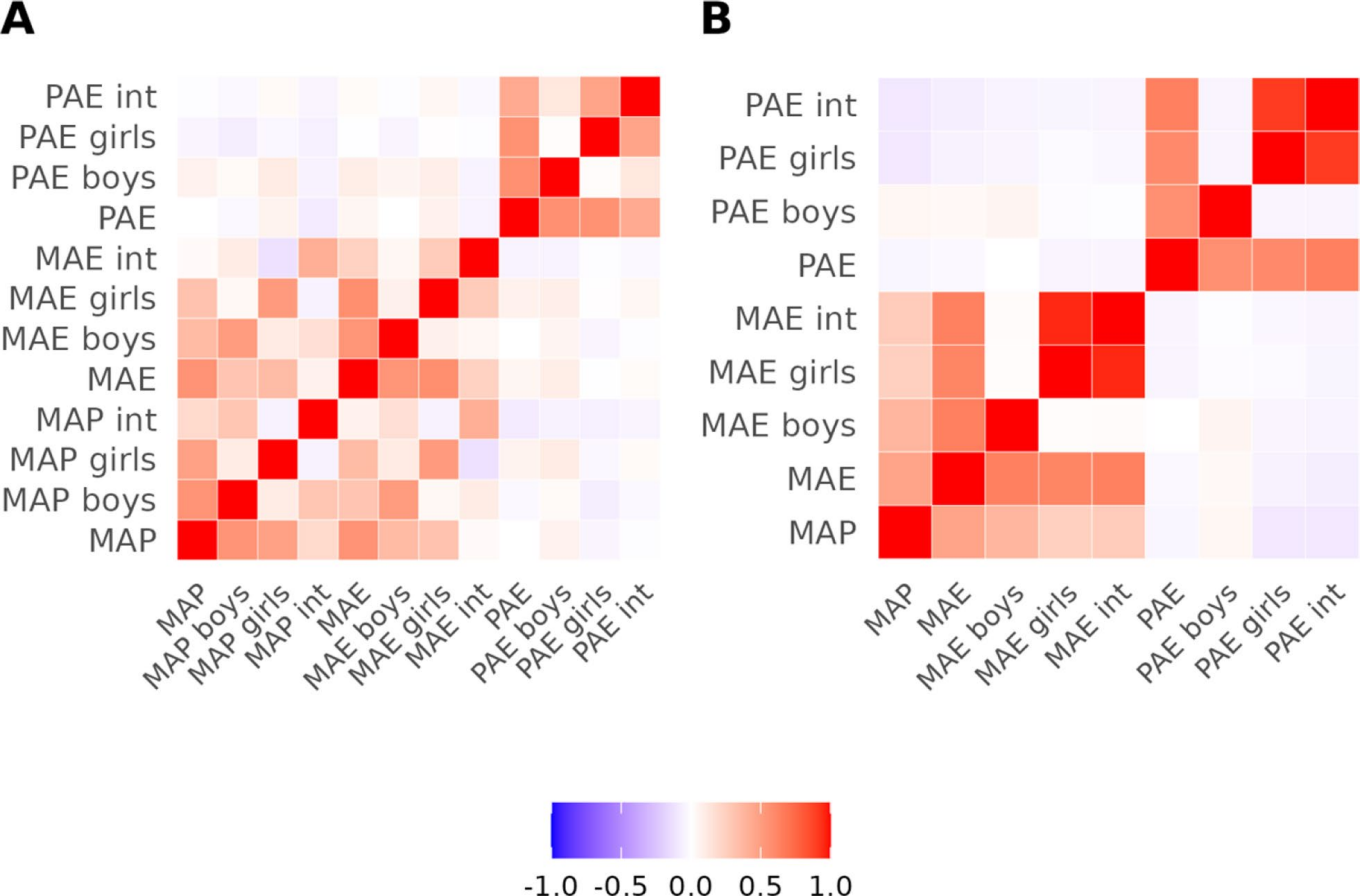
Spearman correlations of effects from meta-analyses of all included models for A) CpGs pertaining to the overlap between the 450 K and EPIC chip and B) the CpGs unique to the EPIC chip. MAP, maternal asthma during pregnancy; MAE, maternal asthma ever while; PAE, paternal asthma ever. Sex-stratified models are defined by suffixes. ‘Int’ represent interaction models testing for differences between the sexes

We correlated methylation effect sizes across 450 K CpGs from the meta-analyses results from maternal asthma during pregnancy (MAP), maternal asthma ever (MAE), maternal asthma ever without using MAP data (MAE exclusive) as well as paternal asthma ever (PAE) (Fig. 3). We observed a moderately strong positive correlation between MAP and MAE effect sizes (rho = 0.55, *P* value < 0.001), as well as positive correlation between MAP and MAE exclusive effect sizes (rho = 0.47, *P* value < 0.001). We did not observe a correlation between MAP and PAE (rho = 0.002, *P* value = 0.15), but observed a weak positive correlation between MAE and PAE (rho = 0.05, *P* value < 0.001) and p between MAE exclusive and PAE (rho = 0.06, *P* value < 0.001).

For the EPIC chip, MAP and MAE effect sizes we again found moderate positive correlated (rho = 0.47, *P* value < 0.001). MAP and PAE were weakly negatively correlated (rho = − 0.04, *P* value < 0.001) as were MAE and PAE (rho = − 0.03, *P* value < 0.001) (Fig. 3).

The single DMC found on the 450 K chip, cg26963854 located on chromosome 14, that passed the FDR threshold of 5% in maternal asthma during pregnancy had a similar direction of effect across the maternal asthma ever and paternal asthma ever models but was only nominally significantly associated (*P* < 0.05) in the maternal asthma ever meta-analysis.

Of the 26 DMCs identified in maternal asthma during pregnancy meta-analysis on the EPIC chip, we focused on the 21 that were available across the maternal and paternal asthma ever models. We observed similar direction of effect in the maternal asthma during pregnancy and the maternal asthma ever meta-analysis for 19 of these 21 CpGs, and among these 19, we observed 13 nominally significant *p* values (Table S7). In contrast, none of the CpGs identified in the maternal asthma during pregnancy analysis had significant *p* values in the paternal asthma ever model nor consistent directions of effect (Table S7). Of the two DMCs, cg08311378 and cg07462855, identified in paternal asthma ever meta-analysis on the EPIC chip, we did not observe a similar direction of effect when compared to effect sizes in MAP (Table S8). We also did not observe nominally significant *p* values in the MAP meta-analysis results. Compared to the MAE analysis effect estimates, we saw that cg07462855 had a similar direction of effect but neither of the DMCs were nominally significant (Table S8).

### CpGs associated in the literature with childhood asthma or pulmonary function

We uploaded the top CpGs on the 450 K chip identified for MAP (Table S9), MAE (Table S10) and PAE (Table S11) to the EWAS toolkit platform [30] to investigate enrichment in previous DNAm results. For the trait enrichment analyses using MAP-associated CpGs, we observed enrichment in the following traits (which were also among the top10 most associated traits): asthma, smoking, atopy and maternal smoking (all had enrichment *P* values < 1.98 × 10^–13^) (Table S12).

Similarly, we observed that asthma and smoking were the top two traits among all traits when using MAE-associated CpGs (Table S13). Using CpGs associated with paternal asthma ever, we also observed significant association with asthma and smoking (Table S14).

In addition, we investigated enrichment for DMCs identified in the literature specifically for childhood asthma and lung function [8, 11, 31, 32]. We included DMCs identified in blood in an investigation of neonates developing asthma and among children with a clinical diagnosis of asthma in Reese et al. 2019 [8], DMCs in whole blood from childhood asthma in Xu et al. 2018 [31], DMCs identified in nasal epithelial cells from Qi et al. 2020 [32] and unique DMCs identified across DMRs for FEV_1_, FEV_1_/FVC and FEF_75_ in cord blood [11]. In total, 766 previously identified DMCs were used as the enrichment target, and we considered significant enrichment using a *P* value cutoff of 5% from Fisher’s exact test and used as input the CpGs in our analyses with *P* values below 0.005 (Table S9, S10 and S11). We did not observe any enrichment across maternal asthma during pregnancy (*P* value = 0.57), maternal asthma ever (*P* value = 0.84) or paternal asthma ever (*P* value = 0.85).

### Sex-specific analyses

Sex-stratified analyses were performed for all 3 main phenotypes. For the sex-stratified meta-analysis of newborn methylation and maternal asthma during pregnancy, we included 102/943 exposed/non-exposed for boys and 83/781 exposed/non-exposed for girls from 3 cohorts: MoBA1, MoBA2 and NorthPop. We observed 6 DMCs for boys and 325 DMCs for girls related to maternal asthma during pregnancy at FDR < 0.05. None of the 6 DMCs identified in boys gave evidence of significant interaction with sex (FDR < 0.05) (Table S15. Investigating the 325 DMCs identified for girls, 154 had nominal significance for the sex-specific interaction; however, none was statistically significant (FDR < 0.05). Of these 325, 19 were also nominally significant (14 had same direction of effect) in the boys and not among the 6 DMCs identified in boys alone (Table S16).

For the sex-stratified meta-analysis of newborn methylation and maternal asthma ever, we included 309/2347 exposed/non-exposed for boys and 278/2206 exposed/non-exposed for girls from 7 cohorts: ALSPAC, GENR, LiNA, MoBA1, MoBA2, NorthPop and UpstateKIDS; we identified 1 DMC in boys and 25 in girls; none gave evidence of interaction with sex (FDR < 0.05) but 15 of the DMCs identified for girls met nominal significance (Table S17). For paternal asthma exposure, we included 183/1920 exposed/non-exposed for boys and 182/1834 exposed/non-exposed for girls from 7 cohorts: ALSPAC, GENR, LiNA, MoBA1, MoBA2, NorthPop and UpstateKIDS, and we observed 100 DMCs in boys and 95 DMCs in girls (Table S18, S19). Among the 100 DMCs identified in boys only, we saw nominally significant evidence of interaction for 5 sites (Table S18). Among the 95 DMCs found in girls, we observed ten DMCs, three with known annotation near genes *HCCA2*, *C1orf198* and *PNMT*, with statistically significant interaction (FDR < 0.05) (Table S19). For meta-analysis of sex interaction for paternal asthma exposure, we identified 12 DMCs (Table S20), where we observed in general lower methylation and a stronger effect in girls compared to boys.

### Differential DNA methylation and gene expression in blood and lung

To investigate whether differently methylated sites may be associated with gene expression, we analyzed eQTM pairs for 832 blood samples available from the HELIX consortium for child blood [29] and eQTM pairs for 424 lung samples from the GTEx consortium [33].

Among the 402 DMCs identified across all models using the 450 K chip, we observed 15 unique DMCs with significant associations with gene expression in blood (Table S21). Among these associations, we observed a decreased expression of *FAM43A* with increased methylation of cg02072170*,* and this gene is associated with eosinophil counts and thus associated with asthma etiology [34]. We also observed increased expression of *LTBP1* with increased cg15772133 methylation, and this gene has been associated with FEV_1_/FVC in adults [35, 36].

Among the 441 unique DMCs that are encompassed by identified DMRs across all models using the 450 K chip, we found 188 significant unique methylation and gene expression pairs using the HELIX data (Table S22). We observed CpGs that annotated to genes that previously have been associated with asthma in adults including *HLA* genes [37].

Examining 56 DMCs identified from DMRs in models with maternal asthma during pregnancy and ever as the primary exposures, we found 35 unique DMCs were associated with gene expression of 4 genes. One gene of particular interest is *HOXA5*, which has previously been associated with organogenesis [38], lung function in adults [39, 40] and mental disorder phenotypes [41] (Table S22). The two other identified genes (*KDM2B and KCTD11*) were found to be involved with neurodevelopmental disorders [42] and cancer [43], respectively.

Among the 402 unique DMCs identified across all models using the 450 K chip, we observed one association between increased methylation of cg20810675 and decreased expression of *C4orf27* in lung tissue in GTEx (Table S23). Among the 411 DMCs that are encompassed in DMRs across all models, we identified substantially more associations with gene expression compared to the 402 unique single DMCs identified (Table S24). Specifically, these associations included many *HLA* gene variants and also the *NOTCH4* gene which has been associated with schizophrenia [44], psoriasis [45] and asthma [46].

### Druggable targets

We identified differential methylation in regions related to parental asthma involving the *HOXA5* and *HLA* genes. *HOXA5* is also the target of CHEMBL4224852, a lysine demethylase, which may implicate epigenetic regulation in asthma development. Several *HLA* genes were also identified as targets for drugs, including *HLA-C* (target of CHEMBL4680046) and *HLA-DRB1* (target of CHEMBL2109447).

## Discussion

We investigated parental asthma and newborn DNA methylation using data from 13 cohorts in the PACE consortium and found evidence that parental asthma is associated with differential DNA methylation in newborns. We identified 27 differentially methylated CpGs and 5 differently methylation regions associated with maternal asthma during pregnancy and none for the maternal asthma ever. These results suggest the relative importance of active maternal asthma on offspring methylation patterns. These CpGs were enriched for published associations with asthma and related phenotypes. While we found many more differentially methylated CpGs in girls, no sex interactions were significant at the epigenome-wide level and few reached nominal significance.

### Interpretation

A stronger effect of maternal asthma during pregnancy compared to maternal asthma with respect to numbers of significant DMCs suggests that the timing of the disease exposure is important. In addition, exposure to maternal asthma during pregnancy may suffer from less misclassification that maternal asthma ever, because exposure was more recent and during pregnancy therefore more likely to be physician diagnosed. Also, we cannot rule out that the timing of asthma in the asthma ever exposure may be a relevant factor and thus “dilute” this exposure compared to maternal asthma during pregnancy. Secondly, we cannot rule out that the observed differential methylation is caused by the mother taking asthma medication during pregnancy. However, we also observed a high correlation of effects between the two maternal exposures, suggesting that at least part of the effect can also be seen for an ever diagnosis.

For paternal asthma, findings were enriched for those identified for maternal asthma during pregnancy, but enrichment appeared to be less strong than for the maternal asthma ever exposure- and chip-specific. The two CpGs identified for paternal asthma were also identified as significant in the maternal asthma analyses, indicating that these may represent more general processes not unique to maternal asthma exposure. We note that the power in terms of included cohorts and individuals for the paternal asthma ever was like the analysis of maternal asthma ever, suggesting that the difference in findings is not due to sample size. Taken together, differential DNA methylation was more pronounced if the exposure is active maternal asthma during pregnancy as compared to paternal asthma, substantiating the specificity of the prenatal window of exposure.

Our findings for parental asthma in relation to methylation in newborns were enriched for overlap with those for asthma-relevant traits in prior EWAS. Specifically, we observed enrichment in asthma, smoking and atopy as well as maternal smoking for CpGs identified using maternal asthma during pregnancy, while maternal asthma ever showed enrichment for asthma and smoking. Taken in concert, we also believe that the enrichment in previous asthma-related CpGs, but not CpGs identified specifically for childhood asthma, underlines that the risk induced by maternal asthma exposure pertains more to a T2 type asthma characterized by more inflammation and a later debut [47]. These findings need to be confirmed in future research on maternal health and its impact on child development and disease predisposition.

We also identified differential methylation in regions related to parental asthma involving the *HOXA5* and *HLA* genes. Both genes have been targets for the drug. Altered expression of *HLA* genes has previously been linked to asthma and allergy [37] while perturbed expression of *HOXA5* has been associated with impaired lung function in children [11] and in adults [39, 40] highlighting the importance of future studies investigating their role in asthma pathogenesis. Notably, *HLA* genes and *HOXA5* have also been associated with mental disorders and the latter may be important in development [41].

There is considerable interest in differential impacts of parental factors on child DNA methylation depending on sex where the effect of parental allergy on childhood allergic diseases has been demonstrated to depend on the sex of the child [48]. More DMCs were identified in girls compared to boys, suggesting that girls might be more susceptible to methylation changes from exposure to maternal asthma. However, we did not observe any statistically significant sex interactions in our analyses (P < 0.05). Thus, the findings presented here should be interpreted with caution. Other studies have suggested a differential effect of maternal asthma on female children in terms of microbiome composition [49] on fetal growth [50] and resulting lower birth weight [51]. Future studies would be needed to determine lack of sex-specific effects.

### Strengths and limitations

The major strength of this study is the large sample size, inclusion of multiple cohorts from different populations and the investigation of epigenetic marks in samples collected at the same developmental stage, which enhances the generalizability of our findings. Furthermore, the study adjusted for potential confounders and included cord blood-specific cell-type adjustment [24], which is crucial given that DNA methylation patterns can vary significantly between different cell types. An additional strength of our study is that it used both data from the Illumina MethylationEPIC BeadChip and the Illumina HumanMethylation450K BeadChip, thus increasing the number of CpGs investigated in relation to parental asthma.

The study also has limitations. First, most of the single DMCs were identified in EPIC unique meta-analyses that included only two cohorts. And secondly, none of the 27 identified CpGs has previously been reported to be association with childhood asthma, allergy or lung function in previous large-scale EWAS meta-analyses [8, 11, 31, 32], but we did observe enrichment datasets previously associated with asthma, smoking and atopy. Thirdly, because methylation was measured in various blood sources, the findings may not directly translate to lung tissue, which is a primary organ affected in asthma [52]. However, a previous study showed a high level of agreement between DNAm in blood and bronchial epithelial cells in functional relevant regions [53]. Also, blood for DNA methylation analyses came primarily from cord blood but in one study, newborn blood spots and in another newborn peripheral blood was used. Reference panels are available for cell deconvolution only for cord blood. This could be a source of between study heterogeneity, but we see little evidence for this. Another limitation is that asthma was based on self-reported or, for parental asthma reports in most cohorts and no report on the timing of disease for the parental ever exposure. This may have led to misclassification or noise in the exposure. Finally, to maximize the number of studies and overall sample size for analyses, we focused on probes overlapping between the 450 K and EPIC arrays. However, this approach inherently excludes the few probes present on the 450 K array that are absent on the EPIC array, potentially omitting relevant findings from these non-overlapping probes.

Our findings have important research implications. Identifying differentially methylated CpGs and regions linked to maternal and paternal asthma may improve understanding of asthma mechanisms and reveal potential therapeutic targets if DNAm changes are causal. While no SNPs were associated with the identified DMCs, asthma-related SNPs affecting DNAm cannot be excluded. Stronger findings for maternal asthma during pregnancy highlight the importance of exposure timing in DNAm changes.

Future studies should study the timing of the asthma diagnosis for both maternal and paternal diagnosis in predicting offspring DNAm changes including if both mother and father had asthma at birth and/or conception. Future research should also explore the functional consequences of any DNAm changes and their use as predictive markers for asthma in children. Longitudinal research on the persistence of these patterns and their impact on disease outcomes could provide further insight into DNAm’s role in asthma.

## Conclusion

Our findings suggest that parental asthma is associated with DNA methylation patterns of newborns, with several DMCs and DMRs identified, specifically in relation to maternal asthma during pregnancy.

## Availability of data and materials

Genome-wide meta-analysis results is available in the following link: 10.5281/zenodo.13219057

## Supplementary Information

Additional file 1.
