# Supplementary material for: Maternal asthma and newborn DNA methylation
Source: Clin Epigenetics. 2025 May 10;17:79. doi: 10.1186/s13148-025-01858-4 (PMC12065361; doi:10.1186/s13148-025-01858-4)
Supplement: Supplementary file 1 — Additional file 1. [file 13148_2025_1858_MOESM1_ESM.docx]

## ALSPAC

**Study Population and ethical approval**

Pregnant women resident in Avon, UK with expected dates of delivery 1st April 1991 to 31st December 1992 were invited to take part in the study^1–3^. The initial number of pregnancies enrolled was 14,541 and 13,988 children were alive at 1 year of age. The total sample size for analyses using data collected after the age of seven is 15,447, resulting in 15,658 foetuses. Of these 14,901 were alive at 1 year of age. G0 partners were invited to complete questionnaires by the mothers at the start of the study and they were not formally enrolled at that time. 12,113 G0 partners have been in contact with the study by providing data and/or formally enrolling when this started in 2010. 3,807 G0 partners are currently enrolled. Blood samples from 1018 mother–child pairs (children at three time points and their mothers at two time points) were selected for analysis as part of the Accessible Resource for Integrative Epigenomic Studies (ARIES, <http://www.ariesepigenomics.org.uk/>)^4^. Please note that the study website contains details of all the data that is available through a fully searchable data dictionary and variable search tool (<http://www.bristol.ac.uk/alspac/researchers/our-data/>).

Ethical approval for the study was obtained from the ALSPAC Ethics and Law Committee and the Local Research Ethics Committees. Consent for biological samples has been collected in accordance with the Human Tissue Act (2004). Informed consent for the use of data collected via questionnaires and clinics was obtained from participants following the recommendations of the ALSPAC Ethics and Law Committee at the time.

**Asthma variables and model covariates**

Data were extracted from questionnaire data and obstetric records. Individuals were included in analysis when mothers reported their child’s ethnic background as “white”. Multiple births were excluded.

Maternal asthma ever (no/yes) was self-reported at 32 weeks’ gestation. Paternal asthma/wheezing after child was born (no/yes) was mother-reported when the study child was 8m old. Child sex, gestational age (weeks) and mother's age at pregnancy (years) were available from obstetric records. Lower Maternal education level (no/yes) was self-reported at 18 weeks gestation. Maternal smoking status during pregnancy (never/quit early/smoked most) was derived using self-reports of active smoking at 12, 18 weeks gestation and 8 weeks post-natal.

**DNA methylation procedure**

Following DNA extraction, samples were bisulphite converted using Zymo EZ DNA Methylation™ kits (Zymo, Irvine, CA, USA). Genome-wide DNA methylation was then measured using Illumina Infinium HumanMethylation450 (HM450) BeadChips. The arrays were scanned using an Illumina iScan. Data were preprocessed and normalised using the R package *meffil* ^5^*.* Normalisation utilised the Functional Normalization algorithm^6^ and included the first 10 control probe principal components (PCs). Full details of the preprocessing and normalization of ARIES has been described previously^5^.

**Data analysis**

Analyses were conducted on a complete case basis and where batch factor levels contained at least 2 individuals. Models were run as per the analysis plan with the following modifications: Mode of delivery was not included as a covariate (high number of missing data points and no evidence to suggest it was associated with maternal asthma ever within ALSPAC data). Bisulphite conversion plate was included as a random effect covariate in the analysis to adjust for batch effects.

**Data availability**

Data are available to researchers by request from the Avon Longitudinal Study of Parents and Children Executive Committee (http://www.bristol.ac.uk/alspac/researchers/access/) as outlined in the study's access policy <http://www.bristol.ac.uk/medialibrary/sites/alspac/documents/researchers/data-access/ALSPAC_Access_Policy.pdf>.

**Funding**

The UK Medical Research Council and Wellcome (Grant ref: 217065/Z/19/Z) and the University of Bristol provide core support for ALSPAC. DNA methylation data in the ALSPAC cohort were generated as part of the UK BBSRC funded (BB/I025751/1 and BB/I025263/1) Accessible Resource for Integrated Epigenomic Studies (ARIES, [http://www.ariesepigenomics.org.uk](http://www.ariesepigenomics.org.uk/)). RG and HRE work in the Medical Research Council Integrative Epidemiology Unit at the University of Bristol which is supported by the Medical Research Council and the University of Bristol (MC_UU_00011/5). This

publication is the work of the authors Hannah R. Elliott and Raquel Granell will serve as guarantors for the contents of this paper.

**Acknowledgements**

We are extremely grateful to all the families who took part in this study, the midwives for their help in recruiting them, and the whole ALSPAC team, which includes interviewers, computer and laboratory technicians, clerical workers, research scientists, volunteers, managers, receptionists and nurses. ARIES DNA methylation data used in this analysis was generated in the Bristol Bioresource Laboratory Illumina Facility, University of Bristol.

## CHS

**Study Population and ethical approval**

The Children’s Health Study (CHS) is a population-based prospective cohort study from age 5 onwards in Southern California, which has been described in detail elsewhere ^7^. The study protocol was approved by the University of Southern California Institutional Review Board and informed, written consent and assent were provided by the parents and children respectively. A total of 5341 children were recruited, all of whom were born between 1995 and 1997 and were followed until age 18.

Based on the availability of newborn bloodspots archived by the state of California, a subset of 273 children was selected for a sub-study in which epigenome-wide DNA methylation was assessed in newborn bloodspots. Multiple births were excluded from analyses (7 subjects). The current analyses include the children who had DNA methylation measurements, parental asthma history and covariate data (N=210).

**Asthma variables and model covariates**

Maternal and paternal asthma were assessed via parent-completed questionnaire at study entry when the subjects were around 6 years old. Mothers were asked if a doctor had ever said that this child’s biologic mother or father had asthma. Information on maternal smoking during pregnancy and education were also obtained from parent-completed questionnaires. Child’s sex, maternal age at delivery, gestational age at delivery and delivery type were obtained from California birth certificates. Maternal age was included as a continuous variable. Maternal smoking status during pregnancy, caesarean section and child’s sex were included as dichotomous variables. Maternal educational level was categorized into three groups based on years of education: less than or finished high school, some college or completed college, and some graduate training.

**DNA methylation procedure**

DNA samples from newborn bloodspots collected at delivery and archived by the state of California were extracted using the QiaAmp DNA blood kit (Qiagen Inc, Valencia, CA). DNA samples from whole blood cells were treated with bisulfite using the Zymo EZ DNA MethylationTM kit (Zymo, Irvine, CA) according to manufacturer’s protocol. DNA methylation was then measured using the Infinium HM450 BeadChip assay (Illumina Inc, San Diego, CA) according to standard protocols. The results of the Infinium HumanMethylation450 BeadChip (HM450) were compiled for each locus as previously described and were reported as beta (β) values. (Noushmehr et al, 2010.)

A series of probe and sample-level filtering measures were performed before normalization. All QC was performed in RStudio with the minfi package^8^. First, samples displaying a mean

detection p-value less than 0.05 were removed. Samples with discordant predicted and

observed sex were removed. Next, probes demonstrating detection p-values less than

0.01 across more than 10% of the sample were removed. Cross-reactive probes

identified in Chen et al. and Benton et al.^9,10^ were removed via the xreactive_probes() command in RStudio from the maxprobes package. Quantile normalization was performed, with outlier samples removed, followed by removal of sex and SNP-associated loci. Cell types were estimated with the estimateCellCounts2() from the FlowSortedBlood package using the IDR probes and the “CordBloodCombined” option. This workflow was performed three times for the sex-combined, male, and female analysis. The combined analyses ran successfully, but only a crude version of the sex-stratified male analysis survived – therefore only three model results are provided.

## DCHS

**Study Population & ethical approval**

Drakenstein Child Health Study (DCHS). The DCHS, a population-based birth cohort,has been described previously^11^. Mothers were enrolled prenatally in their second trimester and followed through pregnancy at two primary care clinics serving two distinct populations (predominantly black African ancestry or predominantly mixed ancestry). Mother-child pairs were followed from birth and infants enrolled in the DCHS have been followed since ^11^. All births occurred at a single, central facility, Paarl Hospital. The present study is based on children from the DCHS with DNA methylation data from cord blood, genotyping data, and information on psychopathology factors and covariates.

Ethical approval for human subjects’ research was obtained from the Human Research Ethics Committee of the Faculty of Health Sciences of University of Cape Town (HREC UCT REF 401/2009; HREC UCT REF 525/2012). Written informed consent was signed by each mother on behalf of herself and her infant for participation in this study.

**Asthma variables and model covariates**

Seven default cell types as mentioned in analysis plan. Cord blood cell type composition was predicted using the most recent cord blood reference data set ^12^ and the IDOL algorithm and probe selection ^13^. Batch effects were removed using ComBat from the R package sva ^14^. 5 genetic principal components included in the model to adjust for population stratification

**DNA methylation procedure**

DNA was isolated from cord blood samples that were collected at time of delivery ^15^. DNA methylation was assessed with the Illumina Infinium HumanMethylation450 BeadChips (n=156).

Pre-processing and statistics were done using R 3.5.1 (<https://www.r-project.org/>). Raw iDat files were imported to RStudio where intensity values were converted into beta values. The 450K and EPIC datasets were then combined using the minfi package ^8^ resulting in 316 samples and 453,093 probes. Background subtraction, color correction and normalization were performed using the preprocessFunnorm function ^6^.

Samples were determined to be outliers if detected using two or more of the following methods: detectOutlier function from the lumi package ^16^, Hannum et al. ^17^ method using the locFDR package (<https://cran.r-project.org/package=locfdr>) and both the outlyx and pfilter functions from the watermelon package ^18^. However, no samples we detected in more than one method and so none were removed for this reason. Samples containing maternal blood contamination (n = 33) were removed ^15^. After the completion of pre-processing technical replicates (n = 7) and samples where reported sex did not match sex chromosome methylation signatures (n = 3) were removed leaving a total of 273 samples remaining for downstream analysis.

This dataset contains 59 probes which detect single nucleotide polymorphisms for quality control purposes and so once observed, were removed. Probes with NAs in ≥ 1% of samples or had a detection p value ≥ 1x10-16 in ≥ 1% of samples were removed (n = 10,868). Probes which bind to the sex chromosomes were removed due to the distribution differences observed (n = 9,896). Probes whose sequence contains a SNP either at the CpG site being measured or at the site of the single base pair extension with a minor allele frequency ≥ 1% ^18,19^ were removed (n = 13,598). Autosomal probes which were in silico predicted to non-specifically bind to sex chromosomes in the genome were also removed (n = 9,698) leaving a total of 409,033 probes remaining for downstream analysis ^18,19^.

## EDEN

**Study Population and ethical approval**

The EDEN (Etude des Déterminants pré et post natals du développement et de la santé de l′Enfant) study is a prospective Birth Cohort Study (https://eden.vjf.inserm.fr/), which has been described in detail elsewhere ^20^. Pregnant women seen for a prenatal visit at the departments of Obstetrics and Gynecology of the University Hospital of Nancy and Poitiers before their twenty-fourth week of amenorrhea were invited to participate. Enrolment started in February 2003 in Poitiers and September 2003 in Nancy; it lasted 27 months in each centre. Among eligible women, 55% (n=2002) accepted to participate. The study has been approved by the ethical committees Comité Consultatif pour la Protection des Personnes dans la Recherche Biomédicale, Le Kremlin-Bicêtre University hospital, and Commission Nationale de l’Informatique et des Libertés.

Immediately after delivery, cord blood samples were collected by research midwives from 1367 consenting cohort participants. To prevent any contamination with maternal blood, the cord was doubly clamped immediately after birth (vaginal delivery) or after extraction of the fetus through the uterine incision (elective cesarean section); repeatedly rinsed and venous cord blood serum was sampled between the 2 clamps. Samples were centrifuged within 24 hours of collection. The serum was separated and samples were stored at −80°C.

**Asthma variables and model covariates**

During a clinical visit at the 24^th^-28^th^ week of gestation, the expecting mothers answered a questionnaire about the occurrence of allergic diseases in their family. Mothers were considered to suffer from asthma in pregnancy in case of a positive answer to the question: “*do you suffer from asthma, confirmed by a doctor?”*; paternal asthma was assessed in case of a positive answer to the question: *“does the future father of your child suffer from asthma, confirmed by a doctor?”*

Child sex, maternal smoking status during pregnancy, gestational age, mode of delivery, and maternal age at child at childbirth were included in the models according to the analysis plan. Socio-economic status was categorized in 2 groups according to the highest diploma achieved by the mother (primary/secondary education vs. tertiary education). EDEN methylation data were collected in children born to French-speaking mothers in the cities of Poitier and Nancy (France). All children had at least one Caucasian European parent. Houseman’s method ^21^ was used including the new combined reference panel ^12^, using the IDOL algorithm. Seven cell types were imputed and included in the analyses: nRBC, CD8T, CD4T, NK, Bcell, Mono, Gran. Plate id was included as covariate.

**DNA methylation procedure**

DNA was extracted using the QIAamp blood kit (Qiagen or equivalent protocols), followed by precipitation-based concentration using GlycoBlue (Ambion). DNA concentration was determined by Nanodrop measurement and Picogreen quantification. 500 ng of DNA was bisulphite-converted using the EZ 96-DNA methylation kit (Zymo Research), following the manufacturer’s standard protocol. After verification of the bisulphite conversion step using Sanger Sequencing, genome-wide DNA methylation was measured using the Illumina Infinium HumanMethylation450 BeadChip. After normalization of the concentration, the samples were randomized to avoid batch effects, and all paired samples were hybridized on the same chip. Standard male and female DNA samples were included in this step as control samples.

DNA methylation data were pre-processed in R with the Bioconductor package Minfi ^8^, using the original IDAT files extracted from the HiScanSQ scanner. Samples that did not provide significant methylation signals in more than 10% of probes (detection *P*=0.01) were excluded from further analysis. Samples were also excluded in cases of low staining efficiency, low single base extension efficiency, low stripping efficiency of DNA from probes after single base extension, poor hybridization performance, poor bisulphite conversion and high negative control probe staining. Further, we used the 65 SNP probes to check for concordances between paired DNA samples from the sample individual and assessed the methylation distribution of the X-chromosome to verify gender. Paired samples with Pearson correlation coefficients <0.9 were regarded as sample mix-ups and were excluded from the study. Probes on sex chromosomes, probes that mapped on multi-loci, the 65 random SNPs assay and probes that contained SNPs at the target CpG sites with a minor allele frequency >10% were excluded during probe filtering ^10^. The allele frequencies of a list of SNPs were obtained from 1000 Genomes, release 20110521 for the CEU population. Finally, to correct Type I and Type II bias, the “DASEN” method was implemented to perform signal correction and normalization ^18^. After quality control, 439,306 autosomal probes remained in EDEN.

## GENR

**Study Population and ethical approval**

The Generation R Study is a population-based prospective cohort study from fetal life onwards in Rotterdam, the Netherlands. Assessments in pregnant women and children consisted of physical examinations, fetal ultrasounds, biological samples, and questionnaires. All children were born between April 2002 and January 2006. The study has been approved by the Medical Ethical Committee of the Erasmus University Medical Center and written consent was obtained from participating parents of their children.

**Asthma variables and model covariates**

Maternal and paternal asthma were defined as “ever asthma” assessed by questionnaire.

Information on maternal age, educational level as measure for socio-economic status, smoking during pregnancy, gestational age and mode of delivery were collected by questionnaires at enrollment. Maternal educational level was categorized as low, middle and high. Information on maternal smoking at enrollment, during pregnancy, and / or midwife or hospital registries was combined with information on maternal smoking thereafter obtained by multiple questionnaires during pregnancy and combined (no; yes). Mode of delivery was categorized as vaginal or cesarean section.

**DNA methylation procedure**

DNA-methylation DNA was extracted from cord blood samples of 979 Caucasian children. Using the EZ-96 DNA-methylation kit (Shallow-well) (Zymo Research Corporation, Irvine, USA), 500 ng DNA per sample underwent bisulfite conversion. Samples were transferred onto 96-well plates in a random order. Samples were processed with Illumina’s Infinium HumanMethylation450 BeadChip (Illumina Inc., San Diego, USA). Quality control of analyzed samples was performed using standardized criteria. Samples were excluded due to sample call rate 1% in the GoNLv4 reference panel were excluded, as were probes with non-optimal binding (non-mapping or mapping multiple times to either the normal or the bisulphite-converted genome), resulting in the exclusion of 49,564 probes, leaving a total of 436,013 probes in the analysis. Data were normalized with DASES normalization using a pipeline adapted from that developed by Touleimat and Tost. DASES normalization includes background adjustment, between-array normalization applied to type I and type II probes separately, and dye bias correction applied to type I and type II probes separately. DASES is based on the DASEN method, but adds the dye bias correction, which is not included in DASEN. Beta-values were calculated for all CpG sites.

## INMA

**Study Population and ethical approval**

The INfancia y Medio Ambiente (INMA) Project is a network of birth cohorts in Spain that aim to investigate the effect of environmental exposure on children’s health ^22^. Criteria for inclusion of the mothers were: (i) to be resident in one of the study areas, (ii) to be at least 16 years old, (iii) to have a singleton pregnancy, (iv) to not have followed any programme of assisted reproduction, (v) to wish to deliver in the reference hospital and (vi) to have no communication problems. Current study uses data from the INMA Sabadell subcohort, that involves mother-child pairs from the Sabadell city, enrolled between 2004-2006. The analysis was restricted to European ancestry children.

The study was approved by the Ethics Committee of the reference hospital, and all participants gave their written informed consent.

**Asthma variables and model covariates**

Information regarding history of maternal and paternal asthma was collected using questionnaires during pregnancy. The questions were the following: Maternal asthma: Has the mother ever had allergic asthma? Paternal asthma: Has the father ever had allergic asthma?. Covariates are the defined as: Maternal age at delivery: self-reported by the mother (expressed as years). Maternal education: Defined based on maternal self-reports and categorized in low/middle (primary or secondary school) and high (university degree or higher). Maternal smoking during pregnancy: Determined by questionnaire and defined as no smoking at any point during pregnancy vs at least some smoking during pregnancy. Child’s sex: Obtained from obstetric records. Gestational age: Gestational age was calculated from the date of the last menstrual period (LMP) reported at recruitment and confirmed using estimates based on the first ultrasound examination (about 12th week of gestation). When the difference between the LMP reported at recruitment and estimated from the ultrasound was ≥ 7 days, we estimated LMP using the crown-rump length^23^. Type of delivery (parto): in INMA was classified in three different levels: (1) vaginal; (2) instrumental; (3) cesarean. For this analysis, two categories were used merging vaginal and instrumental categories. Ancestry: None (restricted to European ancestry children). Selection factor: None. Batch effects: None. Cell type composition: At birth cell type composition was estimated using the “Gervin-Salas” reference set ^24^ in the ‘’FlowSorted.CordBlood.Combined.450K’’ Bioconductor package and normalised using *meffil* R packagen ^25^.

**DNA methylation procedure**

Cord blood was extracted using the Chemagen kit (Perkin Elmer). DNA concentration was determined by NanoDrop spectrophotometer (Thermo Scientific) and with the Quant-iT PicoGreen dsDNA Assay Kit (Life Technologies). Methylation data was produced in two different laboratories as part of two different projects: in the Genome Analysis Facility of the University Medical Center Groningen (UMCG) in Holland, and in the Bellvitge Biomedical Research Institute (IDIBELL, Barcelona). Both laboratories used the recommended Illumina protocol for the Infinium HumanMethylation450 beadchip. Briefly, 500 ng of DNA was bisulfite-converted using the EZ 96-DNA methylation kit following the manufacturer’s standard protocol, and DNA methylation measured using the Illumina Infinium HumanMethylation450 beadchip. DNA methylation data were quality controlled and preprocessed using the minfi package ^25^. A series of steps were completed for quality control and data analysis. The first step was low quality sample removal. First, 2 samples with bad overall quality or with low detection p-value according to the output of the MethylAid package were removed ^26^. Then, we removed 3 samples whose sex was wrongly predicted using shinyMethyl were eliminated ^8^. Following guidelines of Lehne work ^27^, we increased the stringency of the detection p-value threshold to 10E-16 and we filtered 18 samples with a call rate lower than 98%. Data was normalized with the functional normalization method. Correlation between SNPs in replicates replicated samples was checked and probes not measuring SNPs were discarded. 7,136 probes with a call rate lower than 95% were also removed. Probes in sexual chromosomes, crosshibridizing or containing SNPs were flagged but not removed at this point. ComBat was applied to remove laboratory batch effect ^28^. Finally, duplicated samples were removed, prioritizing MeDALL samples over BREATHE samples.

**Funding**

This study was funded by grants from Instituto de Salud Carlos III (Red INMA G03/176; CB06/02/0041; PI041436; PI081151 incl. FEDER funds), Generalitat de Catalunya-CIRIT 1999SGR 00241, Fundació La marató de TV3 (090430), EU Commission (261357-MeDALL: Mechanisms of the Development of ALLergy), and European Research Council (268479-BREATHE: BRain dEvelopment and Air polluTion ultrafine particles in scHool childrEn). We acknowledge support from the Spanish Ministry of Science and Innovation and the State Research Agency through the “Centro de Excelencia Severo Ochoa 2019-2023” Program (CEX2018-000806-S), and support from the Generalitat de Catalunya through the CERCA Program.

## IOWF2

**Study Population and ethical approval**

The Isle of Wight 3^rd^ Generation Cohort (IoW F2, >99% Caucasian) is an ongoing study which recruited 600 newborns of IOW F1 parents from 2010 onwards. Epigenome-wide DNA methylation was measured for 193 children using DNA extracted from cord blood. The National Research Ethics Service Committee South Central - Hampshire B (09/H0504/129) approved the recruitment of the third generation (F2) during pregnancy of their mothers, gave permission to obtain informed consent of mothers and fathers for follow-up assessment of their offspring at 3, 6, 12 months and at 2 years (REC no.14/SC/0133), 3 years (REC no. 14/SC/1191) and 6–7 years (REC no. 17/EM/0083).

**Asthma variables and model covariates**

Maternal asthma (yes/no) were based on questionnaire data collected during pregnancy. Data on both maternal and paternal asthma ever (yes/no) were collected throughout the study, at regular intervals for the mothers and at any time when the fathers were available. Maternal age at delivery was derived from the mother’s date of birth and date of delivery and socioeconomic status (SES) and smoking during pregnancy from questionnaires administered before and during pregnancy. Maternal smoking status during pregnancy was defined as did not smoke or quit early during pregnancy or smoked during most of pregnancy. Maternal SES was grouped into three categories using cluster analysis based on maternal education: 1) left before general certificate of secondary education, 2) completed education at 16 -18 years, 3) Other, e.g., vocational training. Gestational age, the newborn’s sex and mode of delivery data were collected from hospital records. Proportion of seven cord white blood cell types (CD4+ T-lymphocytes, CD8+ T-lymphocytes, NK (natural killer) cells, B-lymphocytes, monocytes, granulocytes nucleated red blood cells- nRBC) were computed by applying the reference-based Houseman method ^21^ using the cord blood reference panel developed by Bakulski et al ^29^ in minfi ^8^.

**DNA methylation procedure**

Bisulphite-converted cord blood genomic DNA samples (n=193) EZ-96 DNA Methylation kit (Shallow) (Zymo Research Corporation, Irvine, USA) was used to measure genome-wide DNA methylation using Illumina Infinium HumanMethylation450 BeadChip (n=130) or Illumina Infinium HumanMethylation EPIC (n=63). Data were pre-processed using the Bioconductor IMA package (Illumina methylation analyzer), quality controlled and methylation values normalised (CPACOR) ^23^. Methylation markers on 65 single nucleotide polymorphism (SNP) and sex chromosomes were removed. Samples were excluded if call rate< 98% and probes set to missing if detection p-values ≥10^-16^. Quantile normalised (limma) intensity values were used to calculate beta values. Batch effects were adjusted for using ComBat ^14,24^, a R package, by including batch as a covariate. Probes common between 450k and EPIC bead chips alone were used in the EWAS.

**Funding**

The third-generation study was funded by the National Institute of Allergy and Infectious Diseases (NIAID) at the National Institute of Health, R01 AI091905 (PI: Wilfried Karmaus). The work of John Holloway is also supported by funds from NIAID/NIH (R01AI121226, MPI: Hongmei Zhang and John Holloway).

## LiNA

**Study Population and ethical approval**

The Lifestyle and environmental factors and their Influence on Newborns Allergy risk (LiNA) study is a running prospective birth cohort study conducted by the Helmholtz Centre for Environmental Research-UFZ in Leipzig, Germany, with the aim to entangle the impact of environmental factors on the maturation of children’s immune system and the development of allergic diseases, obesity, and behavioral disorders. For this study, 629 mother-child pairs were recruited from 2006 until 2008 in the city of Leipzig, Germany. Pregnant women were invited to participate, and after informed consent, maternal blood, and urine samples as well as questionnaire data were collected around the 34th week of gestation. Since birth, children as well as mothers are followed up annually by standardized questionnaires and clinical visits including blood, urine, and feces collection. In maternal and in children’s blood, several immune, metabolome and gene expression analyses have been performed. Methylation analysis (450K) have been performed in cord blood samples (n=472).

All participants gave written informed consent. The LINA study was approved by the Institutional Review Board of the University of Leipzig (046-2006, 160-2008, 160b/2008).

**Asthma variables and model covariates**

Maternal and paternal asthma status ever (yes/no) was defined based on maternal report from the baseline questionnaire around 36 weeks' gestation. Maternal age at delivery was calculated from the mother's date of birth and date of delivery. Mode of delivery and sex of the child were obtained from a short questionnaire after birth. All other mother/child covariate information (socio-economic status, smoking habits, maternal age) was assessed by the mothers in the baseline questionnaire.

Cell type proportions (CD4+ T-lymphocytes, CD8+ T-lymphocytes, NK (natural killer) cells, B-lymphocytes, monocytes, granulocytes nucleated red blood cells) were calculated based on the Houseman method^21^ using the Bakulski reference data set^29^. Proportions were used as additional covariates.

**DNA methylation procedure**

Genomic DNA was isolated from cord blood samples using the QIAmp DNA Blood Mini Kit (Qiagen, Hilden, Germany) followed by bisulfite conversion using the EZ-96 DNA Methylation Kit (Zymo Research Corporation, Orange, USA) according to the manufacturer’s recommendations. All samples subsequently subjected to DNA methylation analyses passed the initial quality control check (n = 472). A genome-wide DNA methylation screen was performed based on the Infinium HumanMethylation450 BeadChip (Illumina, San Diego, USA) array (GPL13534 platform). Data were normalized using the SWAN (subset-quantile within array normalization) method of the minfi R package. DNA methylation values, described as beta values (β), were recorded for each locus in each sample. The β values represent the ratio of methylated signal relative to the sum of methylated and unmethylated signal measured per CpG. Outliers in the β values were removed if they were outside of the 3* IQR range for each CpG. All children with DNA methylation data were of European ancestry.

## MINERvA

**Study Population and ethical approval**

Denmark has a comprehensive neonatal screening program which is used to test for innate errors of metabolism, hypothyroidism, and other treatable disorders. Neonatal blood is collected on standard Guthrie cards and residual material is stored within the Danish Neonatal Screening Biobank. The reason for storing the samples in prioritized order is: (1) diagnosis and treatment of congenital disorders, (2) diagnostic use later in infancy after informed consent, (3) legal use after court order, (4) research projects pending approval by the Scientific Ethical Committee System in Denmark, The Danish Data Protection Agency, and the NBS-Biobank Steering Committee. Thus, research is possible assuming sufficient material remains for the proceeding priorities ^30^. Cases and controls were selected from the iPSYCH case–control sample, which has been recently described ^31^. Briefly, the iPSYCH study population comprises all singletons born in Denmark between May 1st 1981 and December 31st 2005, who are still alive and residing in Denmark at their first birthday and with a known mother. The MINERvA study profiled a subsample of 1800 iPSYCH samples, including an equal number of ASD cases and controls see ^32^ for further details of this cohort.

**Asthma variables and model covariates**

Maternal and paternal asthma exposure was identified based on a combination of an asthma diagnosis (ICD-8: 493, ICD-10: J45-J46) or redeemed at least two prescriptions for asthma medication (ATC: R03AC, R03AK, R03BA, R03DC). Information on diagnoses was obtained from the Danish National Patient Register, and information on redeemed asthma prescriptions was obtained from the Danish National Prescription Registry. We defined two maternal exposure periods: 1) maternal asthma identified based on diagnoses/prescriptions at any point before birth of the child (’Maternal asthma ever before birth’), and 2) maternal asthma identified based on diagnoses/prescriptions during pregnancy (from conception to birth) with the child (‘Maternal asthma during pregnancy’), and one paternal exposure period: paternal asthma identified based on diagnoses/prescriptions at any point before birth of the child (‘Paternal asthma ever before birth’).

All perinatal data used for case–control matching, plus additional information on birth weight and maternal smoking were obtained from the Danish Medical Birth Register or the Central Person Register. Detailed maternal smoking data were used to generate a binary variable indicating whether the mother smoked during pregnancy or not. All diagnoses used for ASD case identification and case/control exclusions were obtained from the Danish Psychiatric Central Research Register (DPCRR) and Danish National Patient Register (DNPR). In Denmark, children and adolescents suspected of ASD or other mental or behavioural disorders are referred by general practitioners or school psychologists to a child and adolescent psychiatric department for a multidisciplinary evaluation, and their conditions are diagnosed by a child and adolescent psychiatrist. Registry reporting is done only by psychiatrists following mandatory training in the use of the World Health Organization International Classification of Diseases (ICD) ^32,33^ .

The MINERvA study was approved by the Regional Scientific Ethics Committee in Denmark and the Danish Data Protection Agency.

**DNA methylation procedure**

Neonatal DNA extractions and DNA methylation quantification were performed at the Statens Serum Institut (SSI, Copenhagen, Capital Region, Denmark), building on a previously described protocol ^34^. Briefly, from each dried blood spot sample two disks of 3.2 mm were used with the Extract-N-Amp Blood PCR kit (Sigma-Aldrich, St. Louis, USA) and eluted in 200 μL buffer. The isolated genomic DNA (160 μL) was converted with sodium bisulfite using the EZ-96 DNA Methylation Kit (Zymo Research, California, USA). DNA methylation was quantified across the genome using the Infinium HumanMethylation450k array (“450 K array”; Illumina, California, USA) and a modified protocol as previously described ^33^. Fully methylated and unmethylated control samples were included on each plate throughout each stage of processing. For further DNA methylation processing and quality control we refer to Hannon et al. ^32^

## MoBa1 and MoBa2

**Study Population and ethical approval**

The Norwegian Mother, Father and Child Cohort Study (MoBa) recruited pregnant women from July 1999 to December 2008^35–37^ .At weeks 17 and 30, women completed questionnaires about general demographic information and previous and present health problems and exposures. MoBa1 is a substudy of MoBa. The substudy was a cohort random sample and asthma cases at age three years ^38,39^ Offsprings in this substudy were born in 2002 to 2004. MoBa2 is a non-overlapping subset selected from MoBa ^39,40^ MoBa2 included a cohort random sample and asthma cases at age seven years and non-asthmatic controls, as well as an additional ~200 subjects who had plasma folate measured. Offspring in this subset were born in 2000 to 2005.

All women who participated in MoBa provided written informed consent. MoBa1 and MoBa2 were approved by the Regional Committee for Ethics in Medical Research in Norway and the Institutional Review Board of the National Institute of Environmental Health Sciences in the USA. Previous MoBa1 and MoBa2 publications were based on data release version 5. These analyses were analyzed using data release version 12.

**Asthma variables and model covariates**

Self-reported parental asthma was collected. Maternal asthma during pregnancy was collected at three time points (17^th^ week of gestation, 30^th^ week of gestation, and 6 months after birth). Women who reported experiencing asthma problems or taking asthma medication during pregnancy were categorized as having asthma during pregnancy. Women who reported experiencing asthma problems or taking asthma medication anytime before birth of the child were classified as having ever asthma. Fathers completed a separate paternal questionnaire during pregnancy. Those who reported ever having asthma problems were classified as ever having asthma.

MoBa participants were linked to the Medical Birth Registry of Norway to collect information on maternal age at delivery, infant sex, mode of delivery, and gestational age. Maternal smoking status was based on the combined questionnaire based on self-reported smoking information at 17^th^ week of gestation, 30^rd^ week of gestation, and 6^th^ month after birth and cotinine data available in all MoBa1 subjects and 221 of the MoBa2 subjects measured in plasma collected at approximately gestational week 18. Cotinine values above 56.8 nmol/L were used to indicate that a mother was smoking at this time point ^41^ Women were categorized as sustained smokers if they smoked past the first trimester of pregnancy or had cotinine levels indicative of a smoker. Women were categorized as non-smokers if they did not report any smoking throughout pregnancy. Women who smoked at the beginning of pregnancy and quit early in pregnancy were categorized as quitters. Maternal pre-pregnancy body mass index was calculated from self-reported weight and height collected at the 17^th^ week of gestation. Maternal socioeconomic status was defined based on self-reported maternal education (less than high school, high school, some college, college and higher). All models were also adjusted for the selection factor. In MoBa1, the selection factor was asthma status at 3 years old (yes/no). In MoBa2, participants were selected into the study because of one of the following three groups: asthma status at 7 years old (yes/no) or had folate measured regardless of asthma status.

Potential confounding effects of blood cell subtypes were estimated by the Houseman method ^42^ with the Gervin et al. combined reference panel for cord blood and the IDOL optimization ^12^.

We used ComBat from the *sva* package in R for batch correction ^24^.

In MoBa1, 29 were missing information on maternal education and 38 were missing covariate information. In MoBa2, 22 were missing information on maternal education and 29 were missing covariate information. Genotype data were measured using Illumina HumanCore. We ran principal components to identify and exclude samples that were ancestry outliers (6 in MoBa1, 2 in MoBa2). This study included newborns with cord blood DNA methylation measurements, maternal education data and covariate data (N=984 from MoBa1; N=632 from MoBa2). Each dataset was analysed separately.

**DNA methylation procedure**

Methylation in cord blood was measured in MoBa1 first ^43^ The same laboratory measured DNA methylation in cord blood in MoBa2 later ^40^.

Details of how DNA methylation were measured and the quality control procedure for MoBa1 and MoBa2 have been previously described ^40^. The same procedures were applied to both studies. Briefly, samples of umbilical cord blood were collected at birth and stored at -80°C. DNA was bisulfite converted using the EZ-96 DNA Methylation kit (Zymo Research Corporation, Irvine, CA). DNA methylation was assessed at 485,577 CpGs using Illumina’s Infinium HumanMethylation450 BeadChip ^44^. We used the *minfi* package in R to read the .idat files and calculate the beta methylation values at each CpG:

$$\beta=\frac{intensity of the methylated allele (M)}{intensity of the unmethylated allele (U) + intensity of the methylated allele (M) + 100}$$

Quality control procedures were applied on the beta methylation values. We excluded 65 control probes, probes on the X chromosome (# CpGs =11,230) and probes on the Y chromosome (# CpGs =416). CpGs missing >10% of methylation values were removed (20 CpGs in MoBa1, 0 CpGs in MoBa2). We excluded samples identified by Illumina to have failed or those with an average detection p-value <0.05 across all probes (49 in MoBa1, 35 in MoBa2), as well as samples with sex mismatches (13 in MoBa1, 8 in MoBa2). We used the beta mixture quantile (BMIQ) to normalize the data ^45^ and used ComBat from the *sva* package in R for batch correction ^24^. Extreme beta methylation values (defined as greater or less than three times the interquartile range) were set to missing.

1,068 samples passed quality control in MoBa1. However, in this data release, 11 participants dropped out of the MoBa1, leaving 1,057 with QC methylation data. In MoBa2, 685 samples passed quality control. 473,844 CpGs were analyzed in MoBa1 and 473,748 CpGs were analyzed in MoBa2.

**Funding**

This research was supported [in part] by the Intramural Research Program of the NIH, National Institute of Environmental Health Sciences (Z01-ES-49019). The Norwegian Mother and Child Cohort Study is supported by the Norwegian Ministry of Health and the Ministry of Education and Research, NIH/NIEHS (contract no. N01-ES-75558), NIH/NINDS (grant no.1 UO1 NS 047537-01) and the Norwegian Research Council/FUGE (grant no. 151918/S10).

## NorthPop

**Study population and ethical approval**

Since 2016, all pregnant women in Västerbotten are invited to participate in the NorthPop Birth Cohort Study together with their partner and child (current pregnancy). Please see NorthPop's homepage for full information on data collection (<https://www.northpop.se/en/home-2/>). Families are followed longitudinally until the child is 7 years old. The data collection includes web-based questionnaires and biological samples. During pregnancy, questionnaires are answered by the mother and the partner. A series of questionnaires are administered when the child is 4 months, 9 months, 18 months, 2 years, 3 years, and 7 years old. Umbilical cord blood is collected in EDTA vacutainer tubes and separated into plasma, buffy coat and erythrocyte fractions using centrifugation. In the current study, DNA was extracted from 200 µL buffy coat samples using the FlexiGene DNA kit (Qiagen), according to the standard protocol, and then eluted and stored in Tris-EDTA buffer. Concentration and purity were determined using NanoDrop (Thermo Fisher Scientific). All samples were handled and stored until analysis at Biobanken norr, Umeå, Sweden. Mothers and their partners were solicited for information. Hence, covariate information was based on self-report. We included 722 newborns with DNA methylation data of whom, 679 were from primipara singleton pregnancies whereas the additional 43 children were from multiple-birth (twin or triplet) pregnancies which included women of different parity (22 primipara 21 multipara).

The NorthPop study was approved by the Research Ethics Committee in Umeå, Sweden, 2014/224-31. Written informed consent was obtained from both parents.

**Asthma variables and model covariates**

Maternal asthma during pregnancy: Have you one of the following allergies (one response was Asthma, coded as 0 if no and 1 if yes) [2-year follow-up questionnaire]. Have you had asthma - yes (1) / no (0) [week 18-24 pregnancy questionnaire]. If both items were answered yes, we assumed that the women have had asthma during pregnancy, otherwise no asthma. Maternal asthma ever

: Have you had asthma - yes (1) / no (0) [week 18-24 pregnancy questionnaire]. Paternal asthma ever:

Have you one of the following allergies (one response was Asthma, coded as 0 if no and 1 if yes) [2-year follow-up questionnaire]. Covariates were the following; Smoking: Have you smoked cigarettes during your pregnancy (No: 0, Yes, I smoked during the last month before I got pregnant: 1, Yes, I smoked during pregnancy:2). (0) and (1) were coded as not having smoked during pregnancy. (2) was coded as quit early if women quit before week 20. (2) was coded as smoked most of pregnancy if women quit after week 20. In addition mothers were asked if the smoke now and if yes, then they were also considered smokers during pregnancy, however few women smoked during pregnancy. Gestational age was expressed as days. Adjustment for batch was done using plate identification.

For maternal socioeconomic status we used maternal education extracted from the Swedish longitudinal integrated database for health insurance and labour market studies (LISA).

**DNA methylation procedure**

Methylation profiling was performed with the Infinium MethylationEPIC BeadChip and the results were analyzed with GenomeStudio 2011.1 from Illumina Inc. All samples included for analysis in this project passed Illumina’s ’Probe call rate’ > 0.98 limit. The ENmix package was used to pre-process the methylation data. Before preprocessing, SNP-related probes, probes with call rate P-value<0.01 and probes with missing in more than 20% of samples were removed. Background correction of methylation signal intensities was made with the ENmix-algorithm. We applied inter-array normalization with the quantile method and probe-type bias adjustment using the RCP (Regression on Correlated Probes) method. Finally, cell counts were estimated using a cord blood reference in minfi and FlowSorted. Blood. EPIC packages. Adjustment for batch was done by including plate identification.

**Funding**

The NorthPop infrastructure receives funding from Västerbotten County Council and Umeå

University (MD and CEW) and has received infrastruture grants from The Kempe Foundations (JCSMK23-0155) and FORTE (2024-01645). DNA extraction, methylation profiling and data analyses were funded by grants from the Swedish Asthma and Allergy Foundation grant number: F2018-0027 (SH), the Swedish Research Council grant number 2019-01187 (SH), the Swedish Heart-Lung Foundation grant number 2020-0473 (SH) and FORMAS, grant number 2021-01098 (SH). The funding bodies had no role in study design, data collection and analysis nor in the preparation of the manuscript.

**Acknowledgments**

We acknowledge all participating families in the NorthPop study; the NorthPop project team for recruitment, follow-up, and blood samplings of study participants; the NorthPop coordinator Richard Lundberg-Ulfsdotter at the Department of Clinical Sciences, Umeå University and the personnel at Biobanken Norr,, Västerbotten county council. Methylation profiling was performed by the SNP&SEQ Technology Platform in Uppsala (www.genotyping.se). The facility is part of the National Genomics Infrastructure (NGI) Sweden and Science for Life Laboratory. The SNP&SEQ Platform is also supported by the Swedish Research Council and the Knut and Alice Wallenberg Foundation.

## UpstateKIDS

**Study population and ethical approval**

Upstate KIDS is a birth cohort of babies born between 2008 to 2010 in New York State (excluding New York City) and was designed to investigate whether fertility treatment is associated with child health outcomes^46^. DNA was extracted from newborn dried blood spots (DBS) from samples originally collected by the New York State Department of Health Newborn Screening Program^47^. Illumina’s Infinium EPIC microarray was used to measure DNA methylation at birth^48^. All subjects provided consent for this secondary analysis.

The New York State Department of Health and the University at Albany (State University of New York) institutional review boards (IRB) approved the study.

**Asthma variables and model covariates**

Maternal asthma during pregnancy was defined based on the ICD-9 codes. Maternal and paternal asthma ever was defined based on maternal report from baseline 4-month questionnaire. Covariate information was also provided by mothers through the baseline questionnaire or through vital records (i.e., infant sex) or combination of both where data was missing from either sources (i.e., maternal education, maternal smoking, mode of delivery).

**DNA methylation procedure**

Methylation data were processed using the minfi package in R. Quantile normalization was used to normalize beta values between two types of probes. A detection *P*-value > 0.01 was used to identify methylation measures which failed detection. β values were replaced as missing if they failed detection or had bead counts < 3. Betas was trimmed using the 3IQR trimming method.

Robust linear regression models were conducted using the sample codes. Only "White" ethnic population, singletons, subjects with consent of this secondary analysis, and complete case analysis were used for the RLM analyses.

**Funding**

Supported by the Intramural Research Program of the Eunice Kennedy Shriver National Institute of Child Health and Human Development (NICHD; contracts #HHSN275201200005C, #HHSN267200700019C, #HHSN275201400013C, #HHSN275201300026I/27500004, #HHSN275201300023I/27500017).

**Acknowledgments**

We thank the Upstate KIDS participants and staff for their important contributions. This work utilized the computational resources of the NIH HPC Biowulf cluster (http://hpc.nih.gov).

## References

1. Fraser, A. *et al.* Cohort Profile: the Avon Longitudinal Study of Parents and Children: ALSPAC mothers cohort. *Int. J. Epidemiol.* **42**, 97–110 (2013).

2. Boyd A, Golding J, Macleod J, Lawlor DA, Fraser A, Henderson J, Molloy L, Ness A, Ring S, Davey Smith G. Cohort Profile: The ‘Children of the 90s’; the index offspring of The Avon Longitudinal Study of Parents and Children (ALSPAC). *International Journal of Epidemiology* **42**, 111–127 (2013).

3. Northstone K, Ben Shlomo Y, Teyhan A et al. The Avon Longitudinal Study of Parents and children ALSPAC G0 Partners: A cohort profile [version 1; peer review: awaiting peer review]. *Wellcome Open Res* **8**, (2023).

4. Relton, C. L. *et al.* Data resource profile: Accessible resource for integrated epigenomic studies (ARIES). *Int. J. Epidemiol.* **44**, 1181–1190 (2015).

5. Min, J. L., Hemani, G., Davey Smith, G., Relton, C. & Suderman, M. Meffil: efficient normalization and analysis of very large DNA methylation datasets. *Bioinformatics* **34**, 3983–3989 (2018).

6. Fortin, J.-P. *et al.* Functional normalization of 450k methylation array data improves replication in large cancer studies. *Genome Biol.* **15**, 503 (2014).

7. McConnell, R. *et al.* Traffic, susceptibility, and childhood asthma. *Environ. Health Perspect.* **114**, 766–772 (2006).

8. Aryee, M. J. *et al.* Minfi: a flexible and comprehensive Bioconductor package for the analysis of Infinium DNA methylation microarrays. *Bioinformatics* **30**, 1363–1369 (2014).

9. Benton, M. C. *et al.* An analysis of DNA methylation in human adipose tissue reveals differential modification of obesity genes before and after gastric bypass and weight loss. *Genome Biol.* **16**, 8 (2015).

10. Chen, Y.-A. *et al.* Discovery of cross-reactive probes and polymorphic CpGs in the Illumina Infinium HumanMethylation450 microarray. *Epigenetics* **8**, 203–209 (2013).

11. Zar, H. J., Barnett, W., Myer, L., Stein, D. J. & Nicol, M. P. Investigating the early-life determinants of illness in Africa: the Drakenstein Child Health Study. *Thorax* **70**, 592–594 (2015).

12. Gervin, K. *et al.* Systematic evaluation and validation of reference and library selection methods for deconvolution of cord blood DNA methylation data. *Clin. Epigenetics* **11**, 125 (2019).

13. Koestler, D. C. *et al.* Improving cell mixture deconvolution by identifying optimal DNA methylation libraries (IDOL). *BMC Bioinformatics* **17**, 120 (2016).

14. Leek, J. T., Johnson, W. E., Parker, H. S., Jaffe, A. E. & Storey, J. D. The sva package for removing batch effects and other unwanted variation in high-throughput experiments. *Bioinformatics* **28**, 882–883 (2012).

15. Morin, A. M. *et al.* Maternal blood contamination of collected cord blood can be identified using DNA methylation at three CpGs. *Clin. Epigenetics* **9**, (2017).

16. Du, P., Kibbe, W. A. & Lin, S. M. lumi: a pipeline for processing Illumina microarray. *Bioinformatics* **24**, 1547–1548 (2008).

17. Hannum, G. *et al.* Genome-wide methylation profiles reveal quantitative views of human aging rates. *Mol. Cell* **49**, 359–367 (2013).

18. Pidsley, R. *et al.* A data-driven approach to preprocessing Illumina 450K methylation array data. *BMC Genomics* **14**, 293 (2013).

19. Price, M. E. *et al.* Additional annotation enhances potential for biologically-relevant analysis of the Illumina Infinium HumanMethylation450 BeadChip array. *Epigenetics Chromatin* **6**, 4 (2013).

20. Heude, B. *et al.* Cohort Profile: The EDEN mother-child cohort on the prenatal and early postnatal determinants of child health and development. *Int. J. Epidemiol.* **45**, 353–363 (2016).

21. Houseman, E. A., Molitor, J. & Marsit, C. J. Reference-free cell mixture adjustments in analysis of DNA methylation data. *Bioinformatics* **30**, 1431–1439 (2014).

22. Guxens, M. *et al.* Cohort profile: The INMA--INfancia y Medio ambiente--(environment and childhood) project. *Int. J. Epidemiol.* **41**, 930–940 (2012).

23. Lehne, B. *et al.* A coherent approach for analysis of the Illumina HumanMethylation450 BeadChip improves data quality and performance in epigenome-wide association studies. *Genome Biol.* **16**, 37 (2015).

24. Johnson, W. E., Li, C. & Rabinovic, A. Adjusting batch effects in microarray expression data using empirical Bayes methods. *Biostatistics* **8**, 118–127 (2006).

25. Güil-Oumrait, N. *et al.* Prenatal exposure to mixtures of phthalates and phenols and body mass index and blood pressure in Spanish preadolescents. *Environ. Int.* **169**, 107527 (2022).

26. Valvi, D. *et al.* Variability and predictors of urinary phthalate metabolites in Spanish pregnant women. *Int. J. Hyg. Environ. Health* **218**, 220–231 (2015).

27. van Iterson, M. *et al.* MethylAid: visual and interactive quality control of large Illumina 450k datasets. *Bioinformatics* **30**, 3435–3437 (2014).

28. Fortin, J.-P., Fertig, E. & Hansen, K. shinyMethyl: interactive quality control of Illumina 450k DNA methylation arrays in R. *F1000Res.* **3**, 175 (2014).

29. Bakulski, K. M. *et al.* DNA methylation of cord blood cell types: Applications for mixed cell birth studies. *Epigenetics* **11**, 354–362 (2016).

30. Nørgaard-Pedersen, B. & Hougaard, D. M. Storage policies and use of the Danish Newborn Screening Biobank. *J. Inherit. Metab. Dis.* **30**, 530–536 (2007).

31. Pedersen, C. B. *et al.* The iPSYCH2012 case–cohort sample: new directions for unravelling genetic and environmental architectures of severe mental disorders. *Mol. Psychiatry* **23**, 6–14 (2018).

32. Hannon, E. *et al.* Elevated polygenic burden for autism is associated with differential DNA methylation at birth. *Genome Med.* **10**, 19 (2018).

33. Mors, O., Perto, G. P. & Mortensen, P. B. The danish Psychiatric Central Research Register. *Scand. J. Public Health* **39**, 54–57 (2011).

34. Hollegaard, M. V., Grauholm, J., Nørgaard-Pedersen, B. & Hougaard, D. M. DNA methylome profiling using neonatal dried blood spot samples: a proof-of-principle study. *Mol. Genet. Metab.* **108**, 225–231 (2013).

35. Magnus, P. *et al.* Cohort profile update: The Norwegian Mother and Child Cohort Study (MoBa). *Int. J. Epidemiol.* **45**, 382–388 (2016).

36. Magnus, P. *et al.* Cohort profile: the Norwegian Mother and Child Cohort Study (MoBa). *Int. J. Epidemiol.* **35**, 1146–1150 (2006).

37. Rønningen, K. S. *et al.* The biobank of the Norwegian Mother and Child Cohort Study: a resource for the next 100 years. *Eur. J. Epidemiol.* **21**, 619–625 (2006).

38. Håberg, S. E. *et al.* Maternal folate levels in pregnancy and asthma in children at age 3 years. *J. Allergy Clin. Immunol.* **127**, 262-264.e1 (2011).

39. Reese, S. E. *et al.* Epigenome-wide meta-analysis of DNA methylation and childhood asthma. *J. Allergy Clin. Immunol.* **143**, 2062–2074 (2019).

40. Joubert, B. R. *et al.* DNA methylation in newborns and maternal smoking in pregnancy: Genome-wide consortium meta-analysis. *Am. J. Hum. Genet.* **98**, 680–696 (2016).

41. Shaw, G. M. *et al.* Mid-pregnancy cotinine and risks of orofacial clefts and neural tube defects. *J. Pediatr.* **154**, 17–19 (2009).

42. Houseman, E. A. *et al.* DNA methylation arrays as surrogate measures of cell mixture distribution. *BMC Bioinformatics* **13**, 86 (2012).

43. Joubert, B. R. *et al.* 450K epigenome-wide scan identifies differential DNA methylation in newborns related to maternal smoking during pregnancy. *Environ. Health Perspect.* **120**, 1425–1431 (2012).

44. Bibikova, M. *et al.* High density DNA methylation array with single CpG site resolution. *Genomics* **98**, 288–295 (2011).

45. Teschendorff, A. E. *et al.* A beta-mixture quantile normalization method for correcting probe design bias in Illumina Infinium 450 k DNA methylation data. *Bioinformatics* **29**, 189–196 (2013).

46. Buck Louis, G. M. *et al.* Methodology for establishing a population-based birth cohort focusing on couple fertility and children’s development, the Upstate KIDS Study. *Paediatr. Perinat. Epidemiol.* **28**, 191–202 (2014).

47. Yeung, E. H. *et al.* Eliciting parental support for the use of newborn blood spots for pediatric research. *BMC Med. Res. Methodol.* **16**, 14 (2016).

48. Yeung, E. H. *et al.* Conception by fertility treatment and offspring deoxyribonucleic acid methylation. *Fertil. Steril.* **116**, 493–504 (2021).
